# Supplementary material for: The role of interfacial donor–acceptor percolation in efficient and stable all-polymer solar cells
Source: Nat Commun. 2024 Feb 8;15:1212. doi: 10.1038/s41467-024-45455-0 (PMC10853271; doi:10.1038/s41467-024-45455-0)
Supplement: Supplementary file 1 — Supplementary Information [file 41467_2024_45455_MOESM1_ESM.pdf]

Supplementary Information

The role of interfacial donor–acceptor percolation in efficient and stable all-polymer solar cells

Zhen Wang,[1] Yu Guo,[1] Xianzhao Liu,[1] Wenchao Shu,[2] Guangchao Han,[2] Kan Ding,[3] Subhrangsu Mukherjee,[3] Nan Zhang,[4] Hin-Lap Yip,[4,5,6] Yuanping Yi,[2] Harald Ade,[3] & Philip C. Y. Chow[1]*

1. Department of Mechanical Engineering, The University of Hong Kong, Pok Fu Lam, Hong Kong SAR, China

2. CAS Key Laboratory of Organic Solids, Institute of Chemistry, Chinese Academy of Sciences, Haidian, Beijing 100190, China

3. Department of Physics and Organic and Carbon Electronics Laboratories (ORaCEL), North Carolina State University, Raleigh, NC 27695, United States

4. Department of Materials Science and Engineering, City University of Hong Kong, Kowloon, Hong Kong SAR, China

5. School of Energy and Environment, City University of Hong Kong, Kowloon, Hong Kong SAR, China

6. Hong Kong Institute for Clean Energy, City University of Hong Kong, Kowloon, Hong Kong SAR, China

*Email: pcyc@hku.hk

Contents

Supplementary Notes

Supplementary Note 1**.** Details of molecular dynamics simulations.

Supplementary Note 2. Binodal profiles of PM6:Y6-SMA and PM6:Y6-PA.

Supplementary Note 3. Stability comparison between Y6-SMA- and Y6-PA-based systems.

Supplementary Figures

Supplementary Fig. 1. Frontier orbitals of acceptors.

Supplementary Fig. 2. Simulated absorption spectra and oscillator strengths.

Supplementary Fig. 3. Molecular structure of PM6.

Supplementary Fig. 4. TA spectra (visible region) of blends.

Supplementary Fig. 5. TA spectra (infrared region) of blends.

Supplementary Fig. 6. Decay kinetics of blended films.

Supplementary Fig. 7. TA spectra (visible region) of neat films.

Supplementary Fig. 8. TA spectra (infrared region) of neat films.

Supplementary Fig. 9. Decay kinetics of neat films.

Supplementary Fig. 10. TA spectra of PM6 film.

Supplementary Fig. 11. TA spectra (visible region) of blends, excited at 550nm.

Supplementary Fig. 12. Decay kinetics of blended films, excited at 550 nm.

Supplementary Fig. 13. TRPL profiles of solutions in CF.

Supplementary Fig. 14. PL lifetime comparison between film and solution samples.

Supplementary Fig. 15. UV-vis absorption spectra of CF solution and film states.

Supplementary Fig. 16. PL spectra of CF solution and film states.

Supplementary Fig. 17. Concentration-dependent TRPL profiles of CF solution samples.

Supplementary Fig. 18. UV-vis absorption spectra of CB solutions.

Supplementary Fig. 19. PL spectra of CB solutions.

Supplementary Fig. 20. Concentration-dependent TRPL profiles of CB solution samples.

Supplementary Fig. 21. Temperature-dependent UV-vis absorption spectra of CB solutions

Supplementary Fig. 22. TRPL profiles of acceptors dispersed in PVK with various fractions.

Supplementary Fig. 23. PLQY, κ_r and κ_{nr} comparisons between film and solution samples.

Supplementary Fig. 24. PL spectra of acceptors dispersed in PVK with various fractions.

Supplementary Fig. 25. PL spectra comparison between pristine acceptor and blended films.

Supplementary Fig. 26. Molecular structure of PY-monomer.

Supplementary Fig. 27. PL spectra and TRPL data for PY-monomer solution and film samples.

Supplementary Fig. 28. TA data for neat PY-monomer film and PM6:PY-monomer blended film.

Supplementary Fig. 29. Decay kinetics for PY-monomer and PM6:PY-monomer blend.

Supplementary Fig. 30. Illustration of simulation process of PM6:Y6 blend.

Supplementary Fig. 31. Illustration of simulation process of PM6:PY-IT blend.

Supplementary Fig. 32. Atom type of repeating unit of PM6 and PY-IT.

Supplementary Fig. 33. Potential as a function of dihedrals of PM6 unit.

Supplementary Fig. 34. Potential as a function of dihedrals of PY-IT unit.

Supplementary Fig. 35. Calculated R_g as a function of equilibration time of PM6 chains.

Supplementary Fig. 36. Calculated end-to-end distances as a function of equilibration time of PY-IT chains.

Supplementary Fig. 37. GIWAXS 2D patterns of neat films.

Supplementary Fig. 38. GIWAXS 2D patterns of blended films.

Supplementary Fig. 39. GIWAXS 1D profiles along in-plane and out-of-plane directions.

Supplementary Fig. 40. R-SoXS profiles of neat films acquired with various energies.

Supplementary Fig. 41. R-SoXS profiles of blended films acquired with various energies.

Supplementary Fig. 42. Molecular structure of D18-Cl.

Supplementary Fig. 43. TA spectra of D18-Cl blended films, excited at 750 nm.

Supplementary Fig. 44. Decay kinetics for D18-Cl blended films.

Supplementary Fig. 45. R-SoXS profiles of D18-Cl blended films.

Supplementary Fig. 46. Estimated binodal curves of PM6:Y6-SMA and PM6:Y6-PA.

Supplementary Fig. 47. Photostability of devices under 1 Sun illumination.

Supplementary Tables

Supplementary Table 1. Summary and comparison of stability of Y6-SMA- and Y6-PA-based systems.

Supplementary Table 2. Summary of exciton lifetimes of acceptor solutions.

Supplementary Table 3. Summary of exciton lifetimes of PVK-dispersed acceptors.

Supplementary Table 4. Summary of κ_r and κ_{nr} along with exciton lifetime and PLQY.

Supplementary Table 5. Summary of the GIWAXS results ((010) peaks along out-of-plane direction).

Supplementary Table 6. Summary of photovoltaic performance of devices.

Supplementary References

Supplementary Notes

Supplementary Note 1. Details of molecular dynamics simulations.

The thin films were simulated with the following procedure (see **Supplementary Figs. 30** and **31**): 1) Constructing two solutions with initial box size of (30×30×30 nm^3). One contains 44 PM6 chains (each with 10 repeating units), 60 PY-IT chains (each with 6 repeating units) and 20000 chloroform molecules; the other one contains 44 PM6 chains, 444 Y6s and 20000 chloroform molecules. 2) Compacting the solution under NPT ensemble at 300 K and 100 bar for 1 ns. 3) 20 ns equilibration under NPT ensemble at 300 K and 1 bar. 4) Simulating solvent evaporation: 200 chloroform molecules were removed every 100 ps. 5) 1 ns equilibration at 300 K. 6) simulation of thermal annealing by heating from 300 to 600 K for 300 ps, then equilibration at 600 K for 300 ps, and cooling from 600 K to 300 K for 300 ps. 7) 20 ns equilibration at 300 K. The results are based on the last 10 ns of the equilibrium process, as the system's energy has already reached equilibrium after the first 10 ns.

The total count of contacting D–A pairs (with contacting atoms more than 6 for each pair) for PM6:Y6 and PM6:PY-IT are calculated to be 1191 and 1178, respectively. Nonetheless, PM6:PY-IT has less acceptor units than PM6:Y6. For comparison, the average number of contacting D–A pairs was calculated by dividing the total count of contacting D–A pairs by the number of acceptor units. This also represents the average neighbors of donor units for each acceptor unit.

The atomic types, bonding, and nonbonding interaction parameters of PM6, PY-IT, Y6 and chloroform molecules are based on the general Amber force field (GAFF). The atomic types of the repeating units of PM6 and PY-IT are defined as shown in **Supplementary Fig. 32**. Among them, atomic charges are obtained by density functional theory (DFT) combined with the

restrained electrostatic potential (RESP) method. Some dihedral angles closely related to molecular configuration are optimized. Potential energy curves for the dihedral angles calculated by DFT at B3LYP/6-311G(d,p) level and GAFF along with the fitted intrinsic torsion potential are shown in **Supplementary Figs. 33** and **34**.

Supplementary Note 2. Binodal profiles of PM6:Y6-SMA and PM6:Y6-PA.

To further understand the blend structural morphology and D–A miscibility, we determine the binodal profiles of PM6:Y6-SMA and PM6:Y6-PA blends from the Flory–Huggins free energy of mixing equation for polymer solutions.[1] The binodal curves of PM6:Y6-SMA and PM6:Y6-PA were calculated with the Flory–Huggins free energy of mixing equation for polymer solutions using the script developed by Enrique Gomez et al.[1] and modified by Ade group. In this case, the molecular weight of PM6 was set to be 100 kDa and the number of repeating units for Y6-PAs was set to be 6 (i.e., molecular weight ~10.8 kDa). Based on Flory-Huggins Theory, the entropy of mixing for polymers *S* can be expressed as

$$\frac{\Delta S}{N} = -k_B\left[\frac{\phi_1}{x_1}ln\phi_1 + \frac{\phi_2}{x_2}ln\phi_2\right]$$

where ΔS is the total change of entropy, *N* is the number of lattice sites, k_B is the Boltzmann constant， ϕ_1 and ϕ_2 is the volume fractions of component **1** and **2** respectively, and x_1 and x_2 is the degree of polymerization of component **1** and **2** respectively. And the energy of mixing can be expressed as

$$\frac{\Delta U}{Nk_BT} = \chi\phi_1\phi_2$$

where ΔU is the total change of energy of mixing, T is temperature, and χ is the Flory-Huggins interaction parameter.

Apply the above expressions to the Helmholtz free energy equation

$$\Delta \mathrm{F} = \Delta \mathrm{U} - \mathrm{T}\Delta S$$

(where ΔF is the total change of free energy), then there is

$$\frac{\Delta F}{Nk_B T} = \chi \phi_1 \phi_2 + \frac{\phi_1}{x_1} ln\phi_1 + \frac{\phi_2}{x_2} ln\phi_2$$

As shown in **Supplementary Fig. 46**, the binodal curves of PM6:Y6-SMA and PM6:Y6-PA blends are significantly different, with the all-polymer blends showing a more symmetric and lower-lying profile, therefore indicating that the morphology of PM6:Y6-PA blends is more miscible than the PM6:Y6-SMA blends (closer to thermodynamic equilibrium).[2] We note that the Flory–Huggins interaction parameter (χ) is widely used as a parameter to compare the D–A miscibility between various OSC blends.[3,4] However, it is important to point out that a direct comparison of the χ values to quantify differences in D–A miscibility is only valid when the two systems show similar binodal profiles.[2] From past research we know that the miscibility between PM6 and Y6-SMA is only ~5% (corresponding to χ of ~3).[5] As can be seen from **S**upplementary Fig. 46, PM6:Y6-SMAs have a large quench depth to the percolation threshold (quench depth denotes how deep a system is located inside the two-phase region of the phase diagram at its initial composition). Given that the miscibility for PM6:Y6-PAs is much greater than 5%, the quench depth for these systems is expected to be significantly smaller than PM6:Y6-SMA, which implies it is easier for Y6-PA-based all-polymer solar cells to be quenched to near the percolation threshold (i.e., larger processing window) and to obtain ideal blend morphology.

While the calculated binodal curves are consistent with our conclusions, we would like to note that, while the binodal determined from the Flory–Huggins free energy of mixing equation for polymer solutions have been proved to accurately represent the morphological property of polymer–NFA blends, relatively few studies have applied this model for understanding the morphology of all-polymer blends, and therefore further research on this topic is needed.

Supplementary Note 3. Stability comparison between Y6-SMA- and Y6-PA-based systems.

It can be observed in **Supplementary Table 1** that only some of the Y6-SMA systems show decent T_{80}, while generally speaking Y6-PA (all-polymer) systems show longer T_{80}. One recent report even shows an extrapolated T_{80} of over 20,000 hours for Y6-PA blend, which implies that with decent molecular modification and device engineering, all-polymer solar cells can achieve superior stability. It should be noted that most the Y6-SMA-based devices show a quick PCE degradation so called “burn-in” at the first a few hours of solar illumination, which comes from the morphology evolution away from the optimal quenched state (close to the percolation threshold).[4] As shown in the device stability data reported in literature and also in this work, such “burn-in” degradation is suppressed in the Y6-PA-based devices compared to those based on Y6-SMAs. This finding is consistent with the conclusion of this work that all-polymer blends based on Y6-PAs show better thermodynamics stability thanks to the increased D-A miscibility and interfacial percolation.

A recent work reported by Huang et al. shows that Y6-PA blends have very good stability. Besides the PCE T_{80} (~2400 h) is more than three times longer than the Y6-SMA blends, in fact the V_{OC} and *FF* are very stable, even more stable than the meanwhile reported best oligomer (OY3).[6] Since V_{OC}/FF are relative to interfacial recombination,[7] this evidence is consistent with our conclusion that Y6-PA blend has the most stable interfacial morphology (thanks to the better

D–A intermixing/percolation). The overall lower (than OY3) T_{80}, as pointed out by Huang et al., is most likely due to the instability of donor hole transport network, as claimed as "the disorder of POY results in the disturbance of crystallinity and orientation of PBDB-T in blend films, being one of the causes for the degradation of J_{SC} in POY-based OSCs".[6]

While thermodynamic stability of the blend morphology is important for the overall OPV device lifetime, other factors may also affect the overall device stability. For instance, Deibel et al. has found that aging-induced defect state formation in the active layer remains the primary cause of thermal degradation and this can be caused by the use of PM6 donor polymers.[8] Gillett et al. reported the critical role of the donor polymer in the stability of organic solar cells, and identified a light-induced twisting in a shared structural motif of PM6 as a dominant degradation mechanism.[9] Most of the recently reported all-polymer systems utilize PM6 as donor and is likely suffering from the instability of donor polymers too. Therefore, besides improving the morphology stability via increased miscibility, it is critical to address these other factors too in order to achieve highly stable and efficient all-polymer OPV devices.

Supplementary Figures

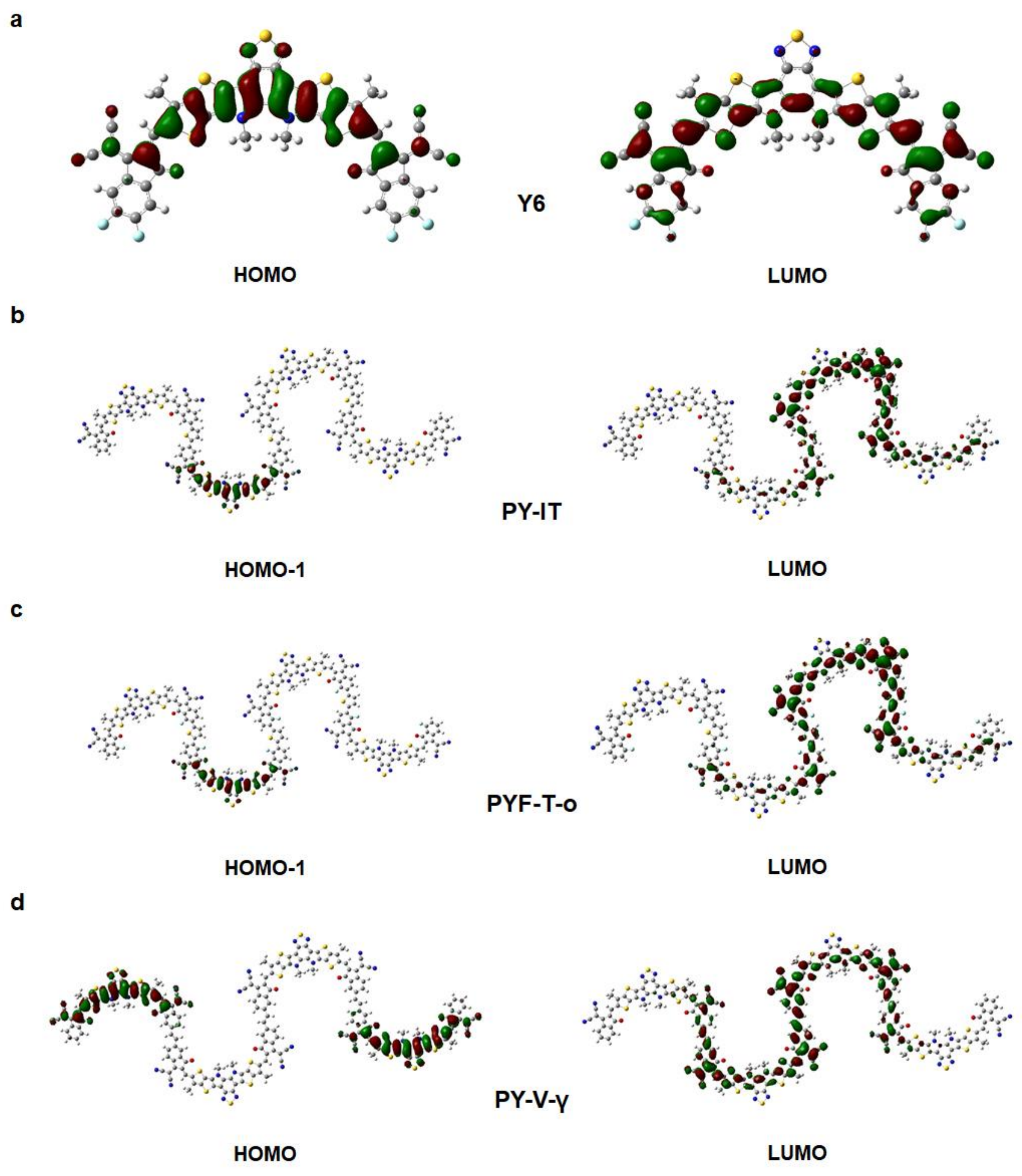

Supplementary Figure 1. Frontier orbitals (only showing the ones that contribute mainly to the S_0-S_1 transition) of Y6 (a), PY-IT (b), PYF-T-o (c) and PY-V-γ (d).

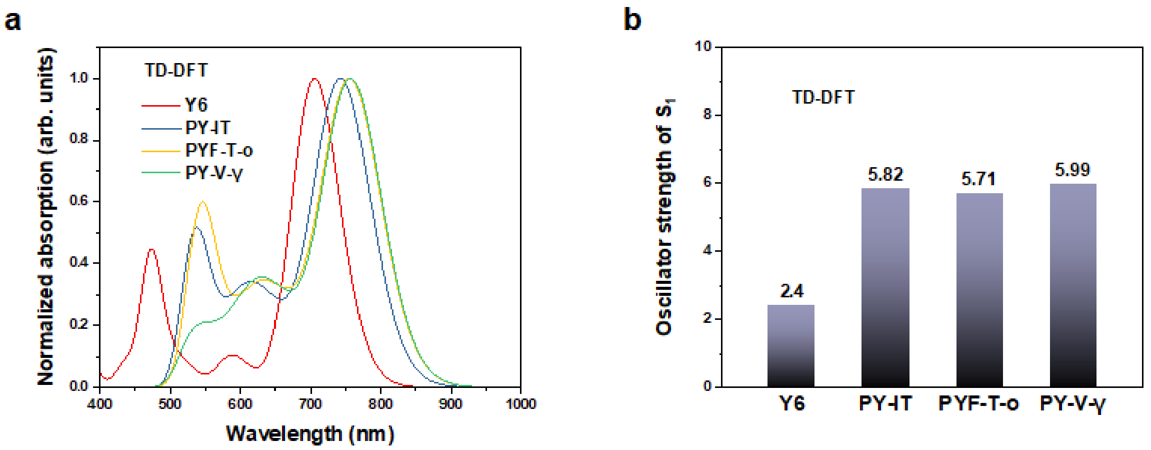

Supplementary Figure 2. Normalized absorption spectra (a) and oscillator strengths of the first excited states (b) calculated with TD-DFT method.

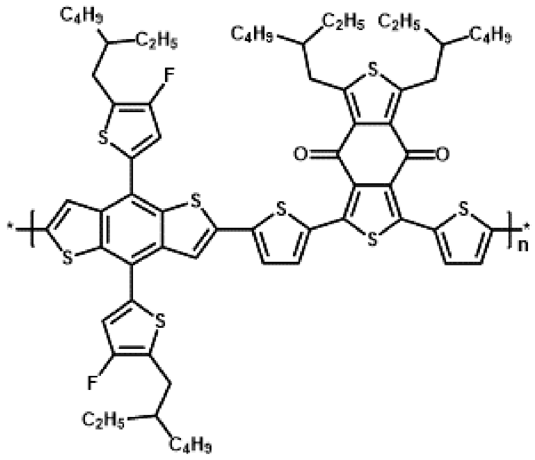

Supplementary Figure 3. Molecular structure of PM6.

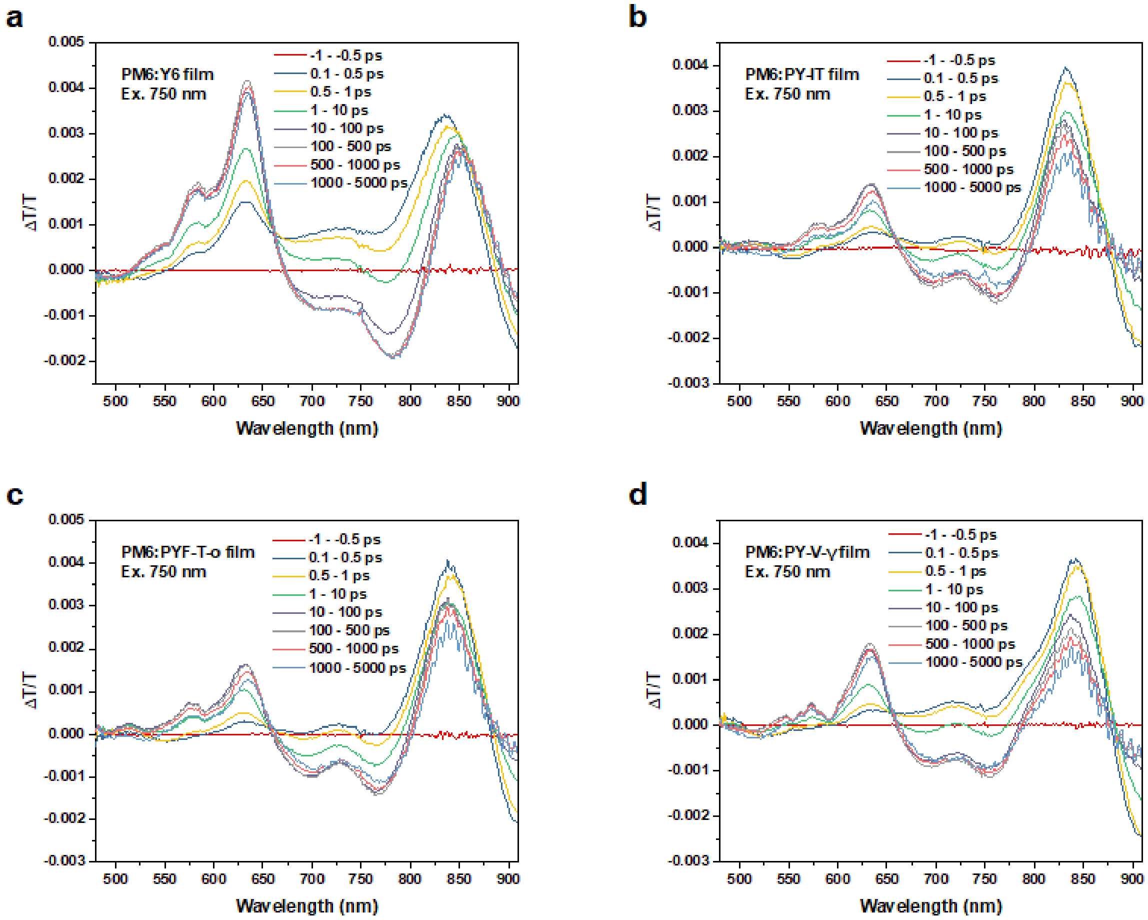

Supplementary Figure 4. TA spectra (visible region) of PM6:Y6 (a), PM6:PY-IT (b), PM6:PYF-T-o (c), and PM6: PY-V-γ (d) blends, excited at 750 nm.

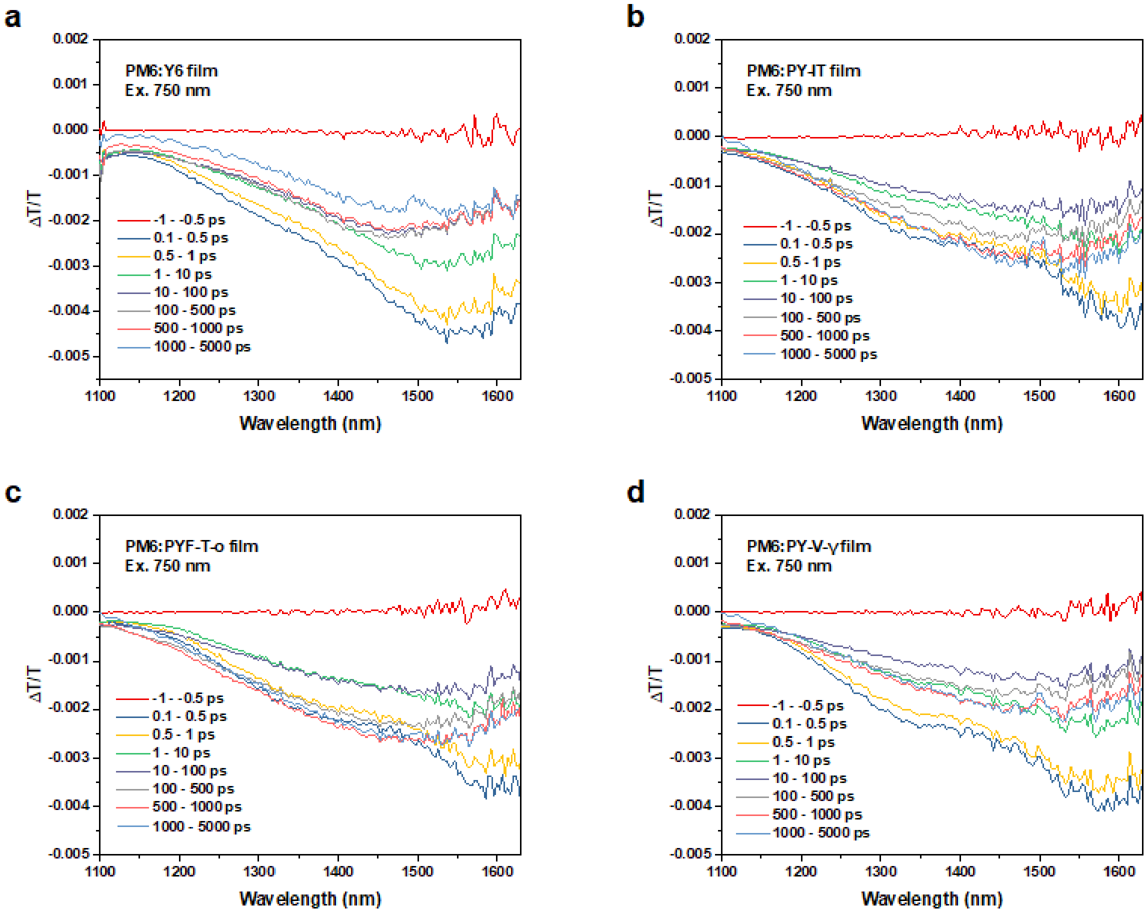

Supplementary Figure 5. TA spectra (infrared region) of PM6:Y6 (a), PM6:PY-IT (b), PM6:PYF-T-o (c), and PM6: PY-V-γ (d) blends, excited at 750 nm.

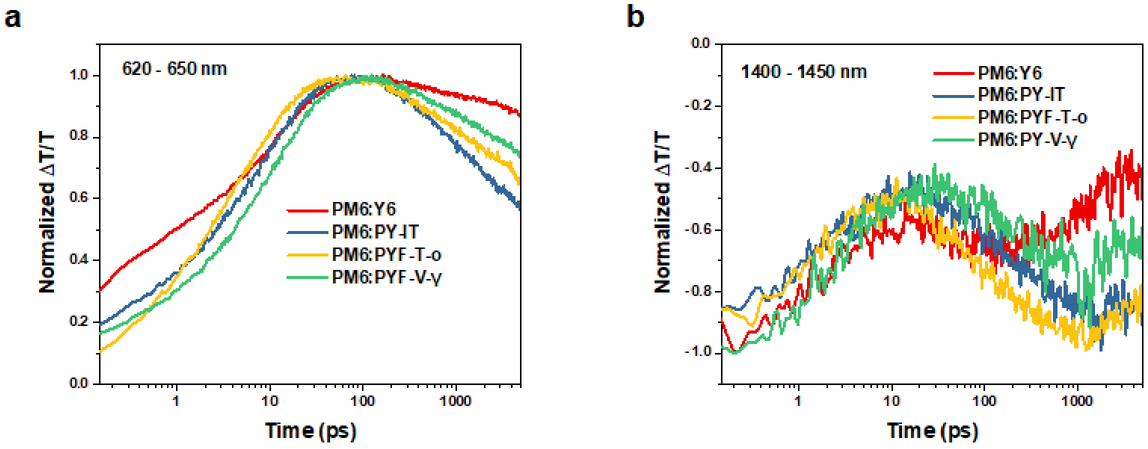

Supplementary Figure 6. Integrated TA kinetics at (a) 620-650 nm and (b) 1400-1450 nm for blended films.

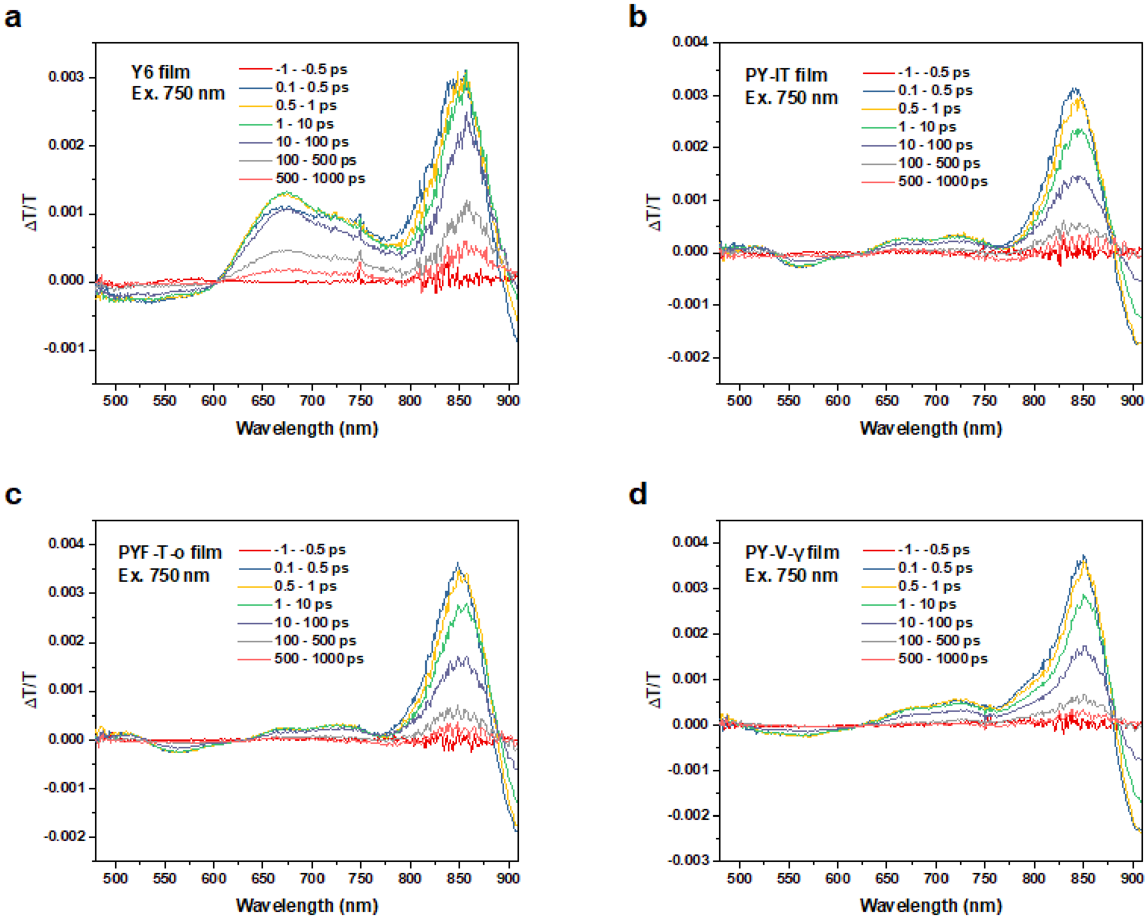

Supplementary Figure 7. TA spectra (visible region) of neat Y6 (a), PY-IT (b), PYF-T-o (c), and PY-V-γ (d) films, excited at 750 nm.

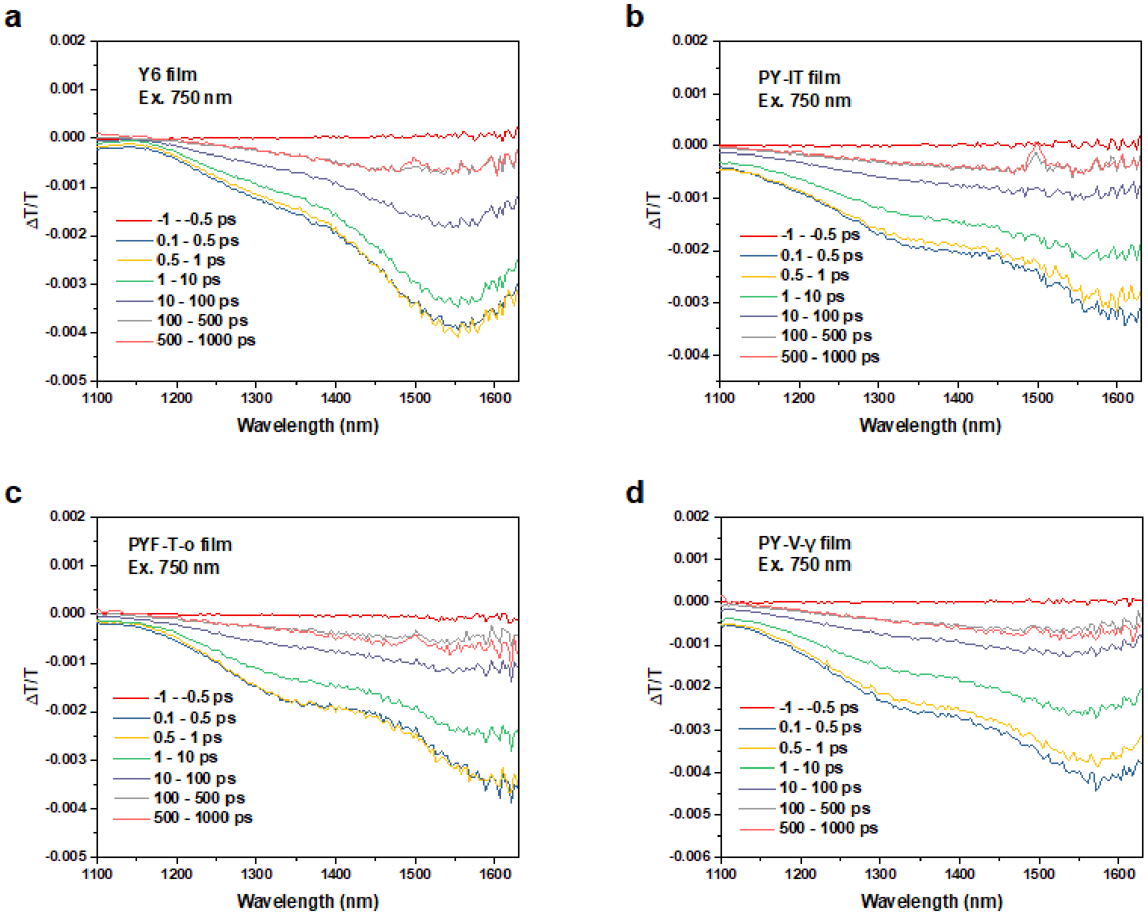

Supplementary Figure 8. TA spectra (infrared region) of neat Y6 (a), PY-IT (b), PYF-T-o (c), and PY-V-γ (d) films, excited at 750 nm.

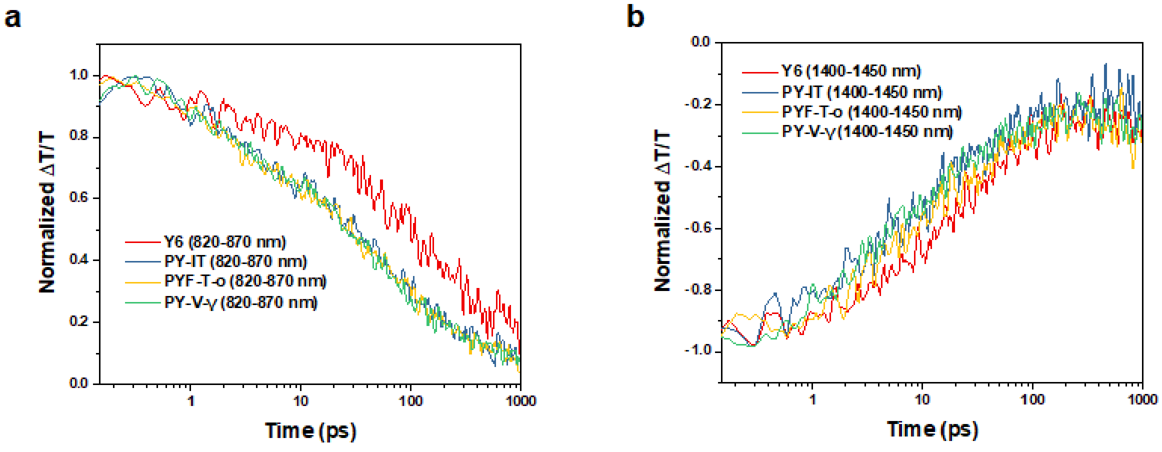

Supplementary Figure 9. Integrated TA kinetics of acceptor ground state bleaching (GSB) feature (a) and polaron absorption (PA) feature (b) in neat films, excited at 750 nm.

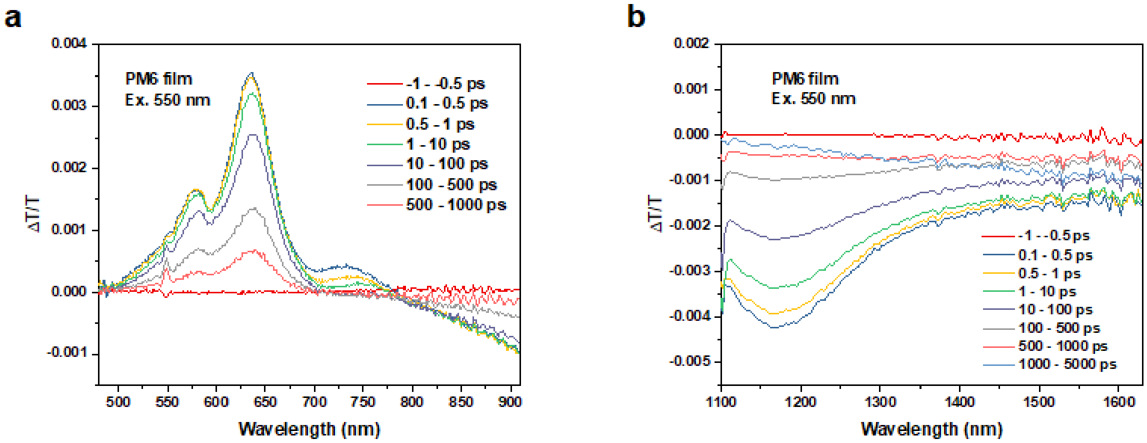

Supplementary Figure 10. TA spectra visible region (a) and infrared region (b) of PM6 film.

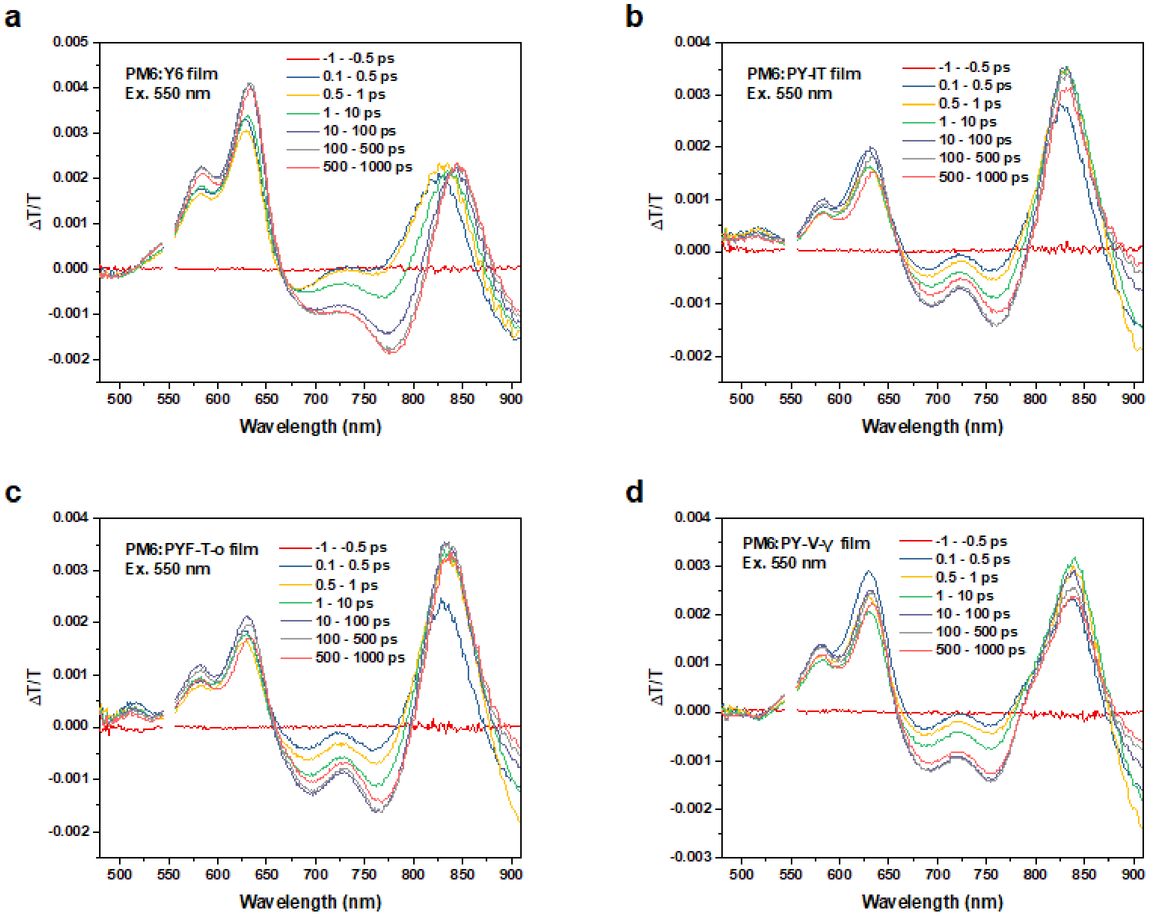

Supplementary Figure 11. TA spectra (visible region) of PM6:Y6 (a), PM6:PY-IT (b), PM6:PYF-T-o (c), and PM6: PY-V-γ (d) blends, excited at 550 nm.

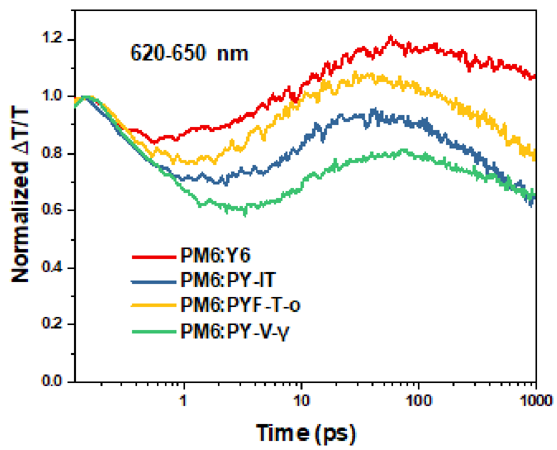

Supplementary Figure 12. Integrated normalized TA kinetics at 620-650 nm for PM6 blended films, excited at 550 nm.

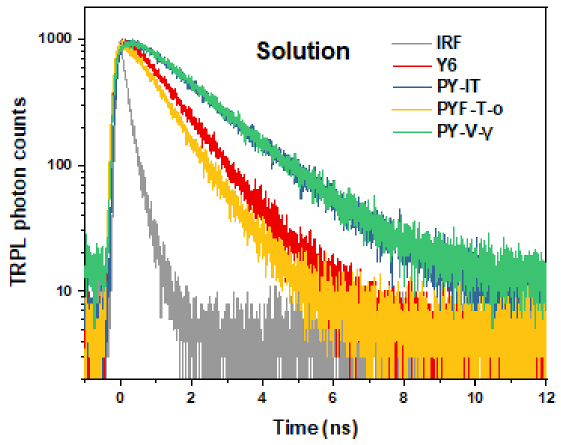

Supplementary Figure 13. TRPL profiles of Y6, PY-IT, PYF-T-o and PY-V-γ solutions in CF (0.03 mg mL^{-1}), excited at 680 nm.

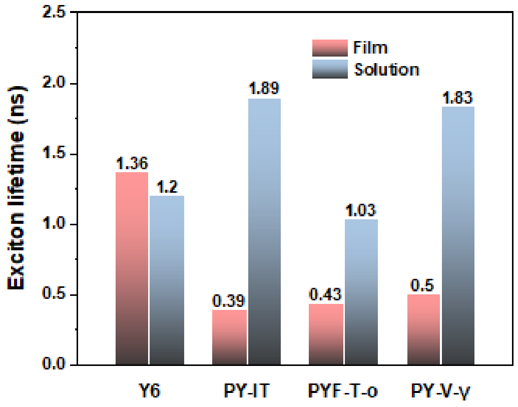

Supplementary Figure 14. PL lifetime comparison between film and solution (0.03 mg mL^{-1}) samples.

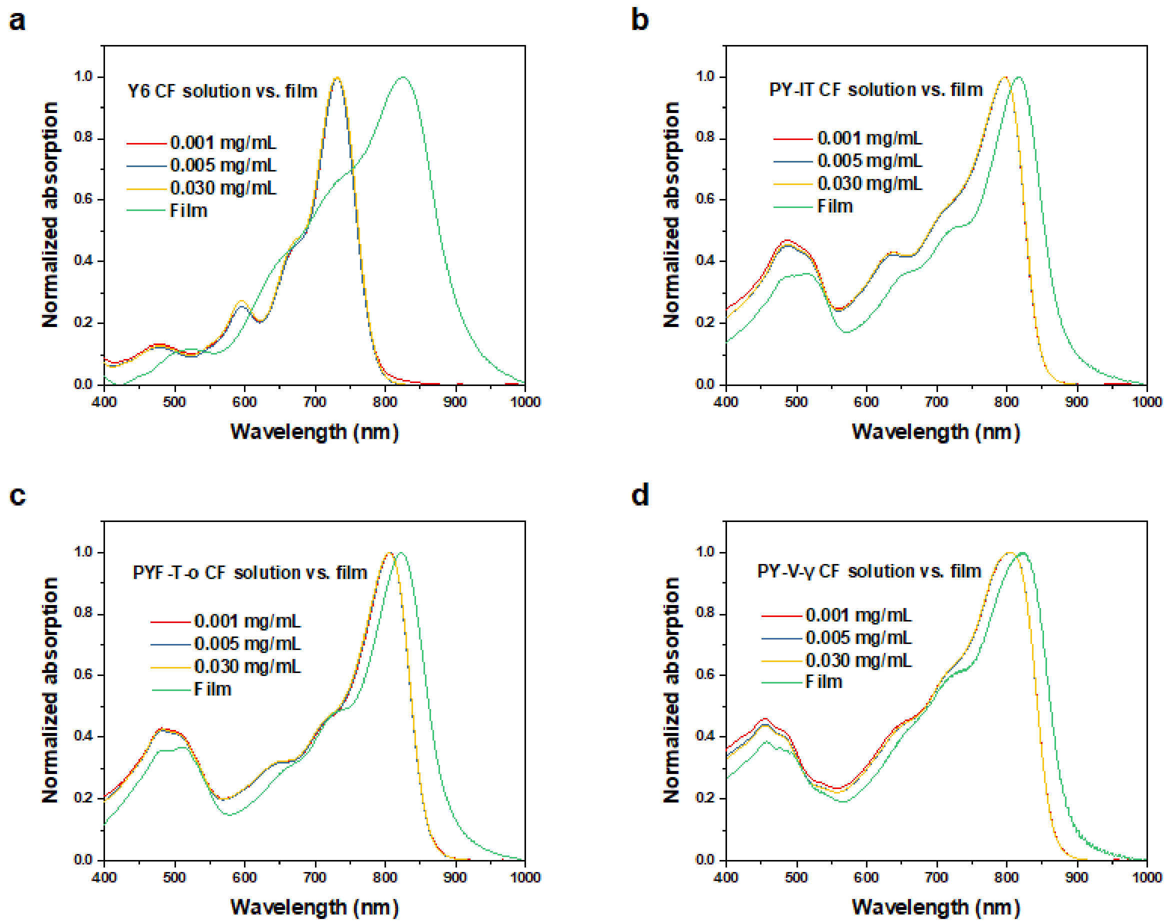

Supplementary Figure 15. Normalized UV-vis absorption spectra of Y6 (a), PY-IT (b), PYF-T-o (c) and PY-V-γ (d) in CF solution and film states.

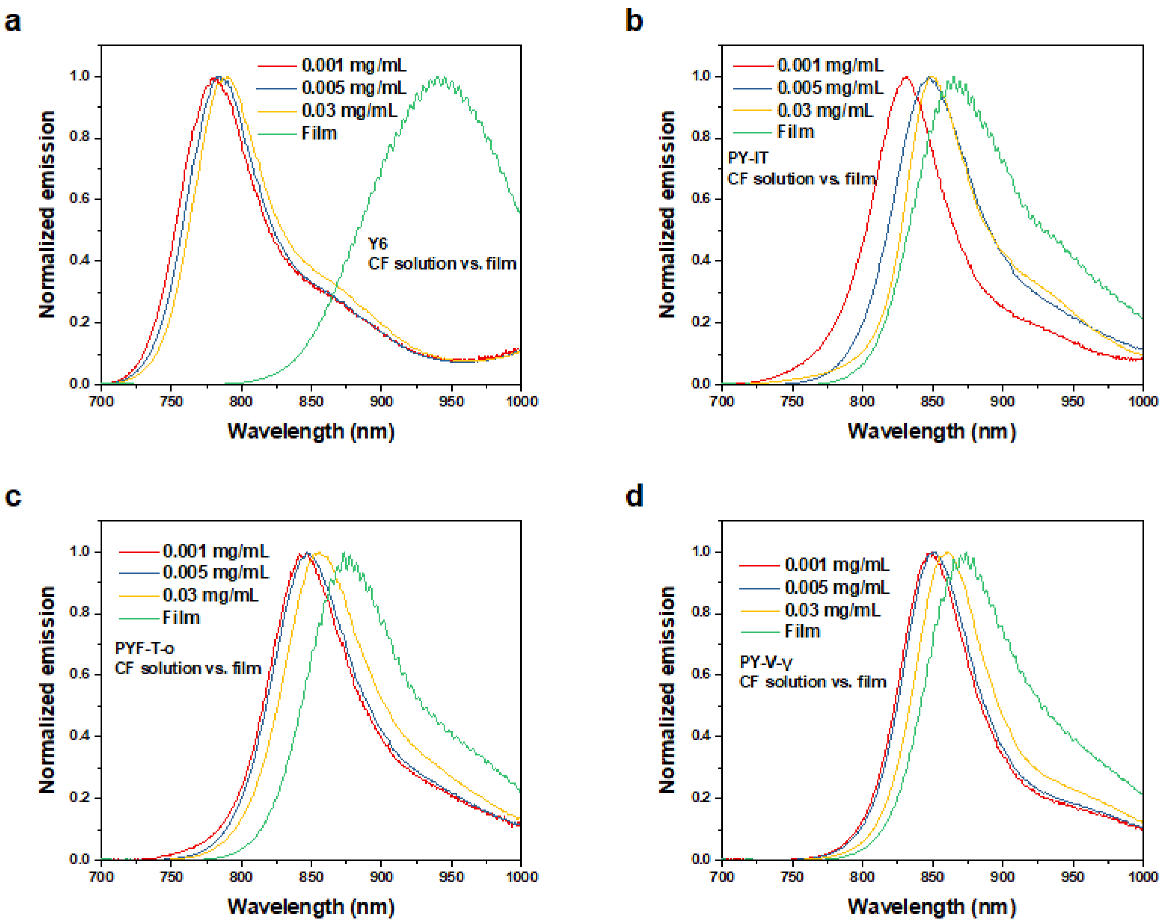

Supplementary Figure 16. Normalized PL spectra of Y6 (a), PY-IT (b), PYF-T-o (c) and PY-V-γ (d) in CF solution and film states.

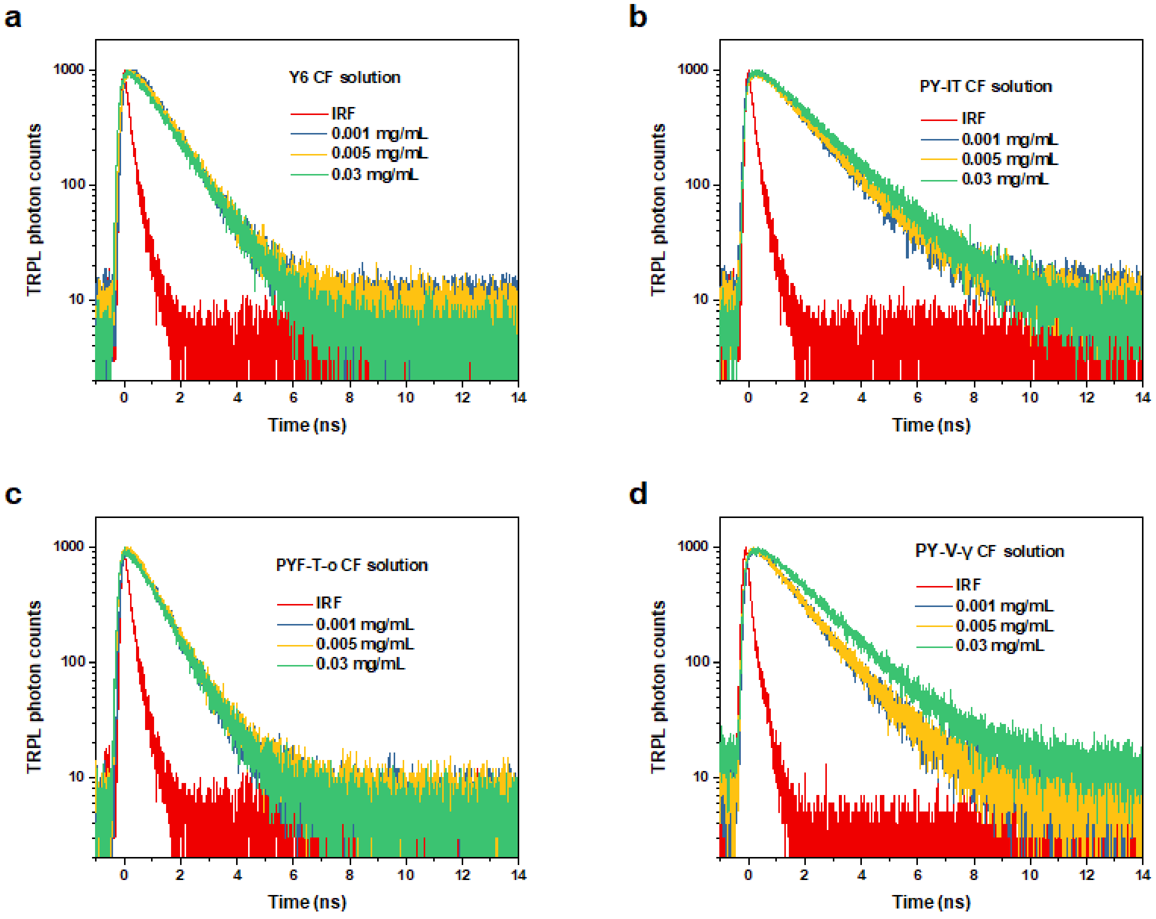

Supplementary Figure 17. Concentration-dependent TRPL profiles of Y6 (a), PY-IT (b), PYF-T-o (c) and PY-V-γ (d) in CF solution.

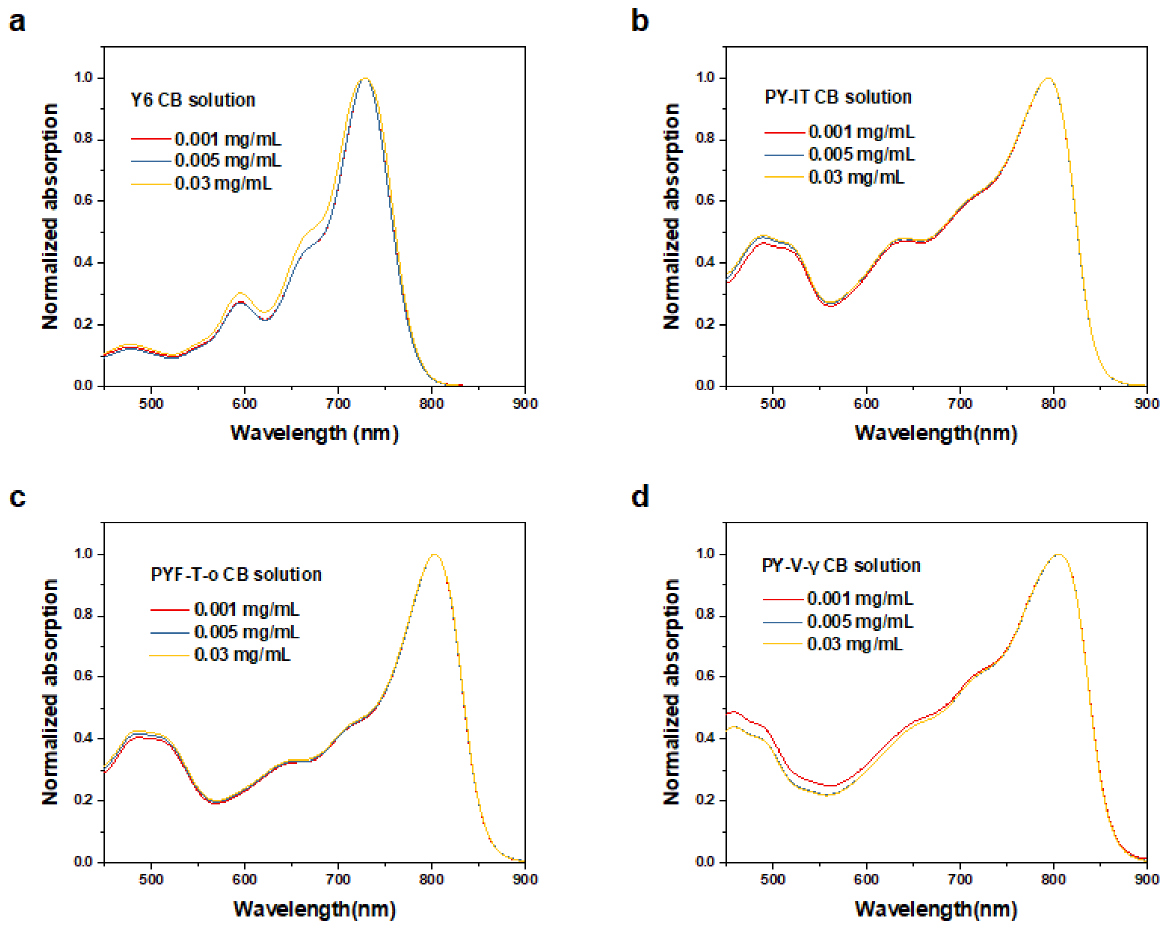

Supplementary Figure 18. Normalized UV-vis absorption spectra of Y6 (a), PY-IT (b), PYF-T-o (c) and PY-V-γ (d) in CB solutions.

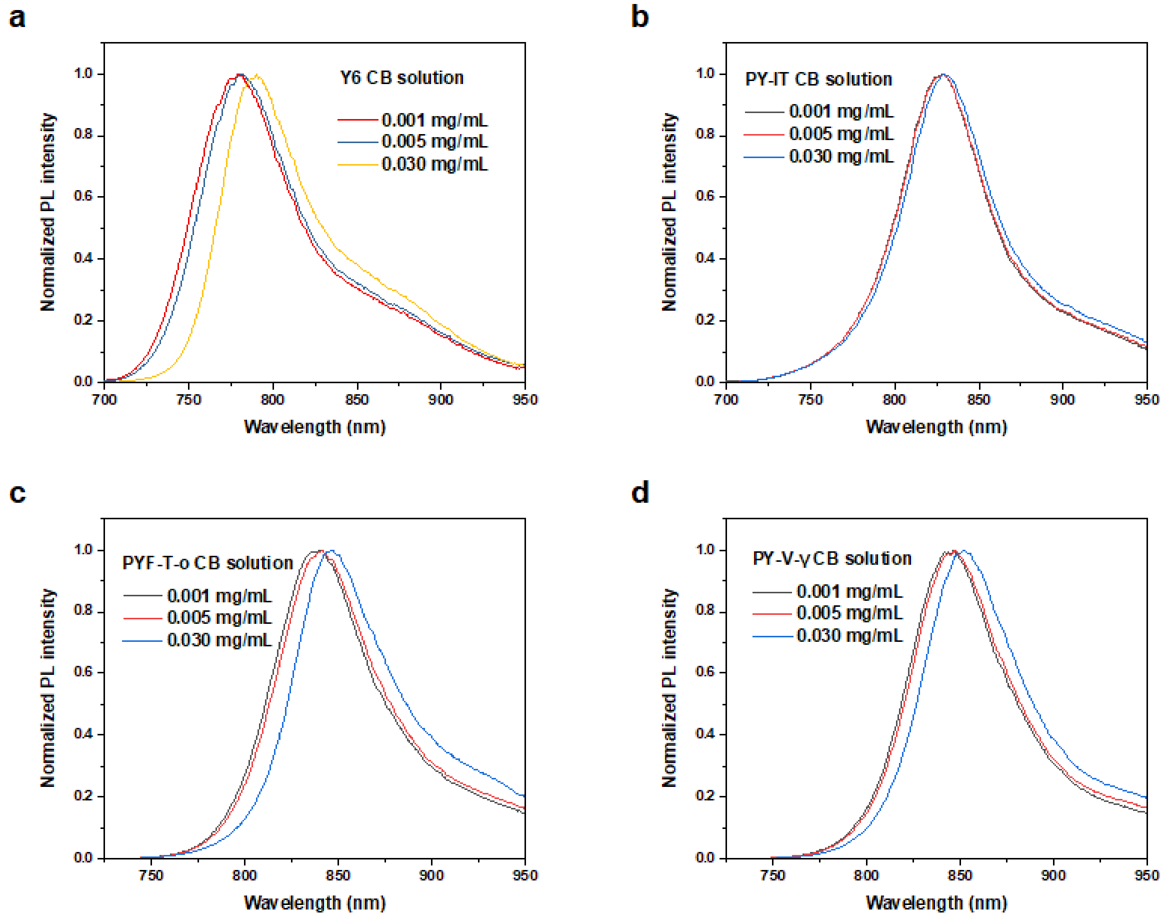

Supplementary Figure 19. Normalized PL spectra of Y6 (a), PY-IT (b), PYF-T-o (c) and PY-V-γ (d) in CB solution.

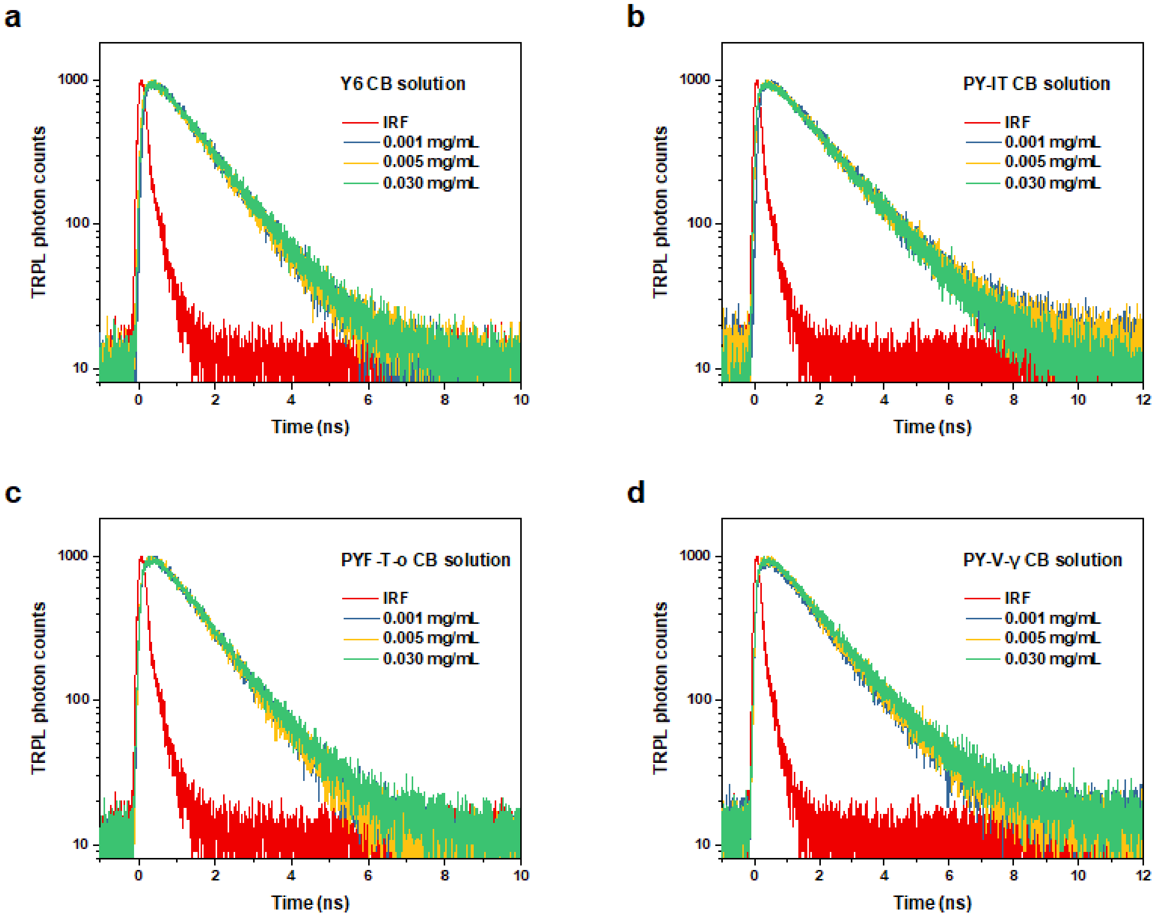

Supplementary Figure 20. Concentration-dependent TRPL profiles of Y6 (a), PY-IT (b), PYF-T-o (c) and PY-V-γ (d) in CB solutions.

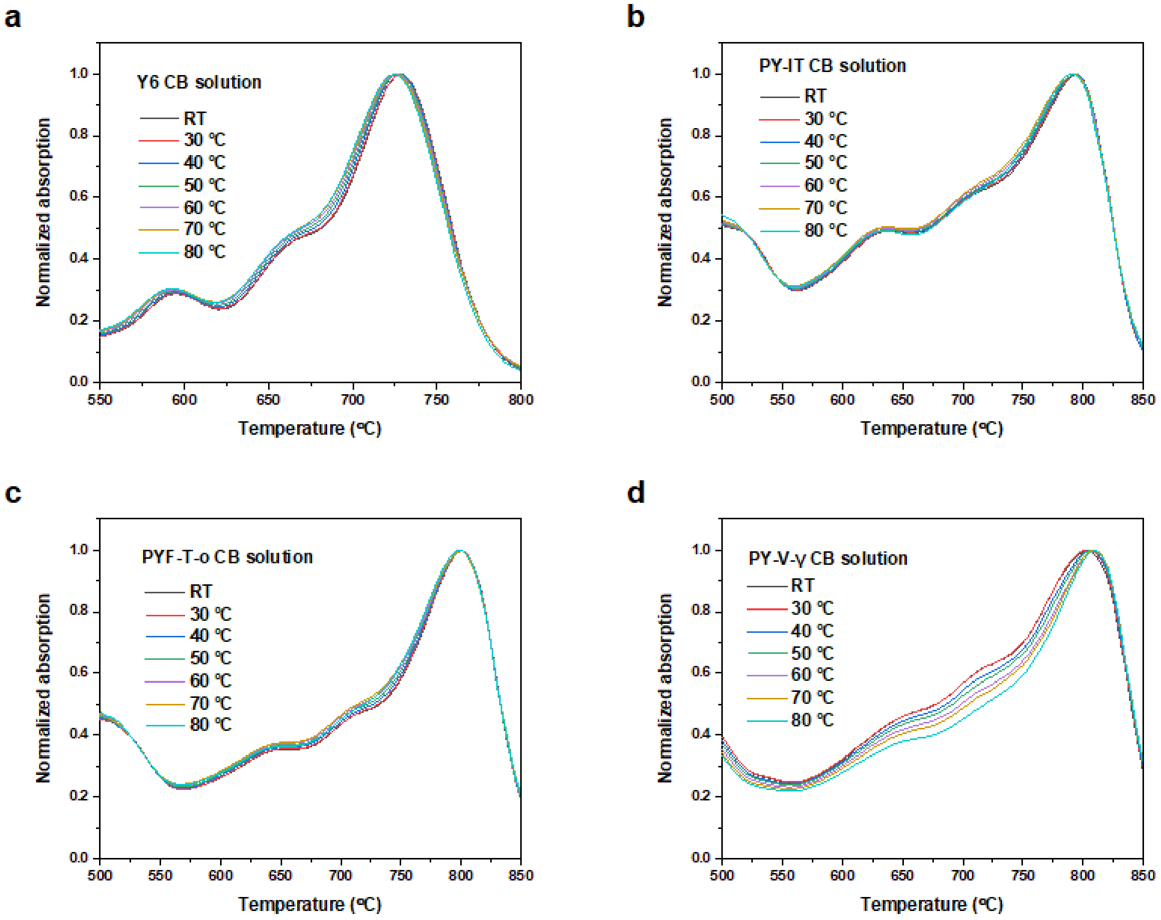

Supplementary Figure 21. Temperature-dependent UV-vis absorption spectra of Y6 (a), PY-IT (b), PYF-T-o (c) and PY-V-γ (d) in CB solutions (0.005 mg/mL).

At higher temperatures, the absorption spectra of solutions samples get broadened due to the stronger electron–phonon couplings and likely molecular relaxation. It is observed that there is slight blueshifts for the solutions as the temperature ramps up, while the shifts are rather small, especially for Y6-PAs. These results therefore indicate that with such low concentration, the degree of aggregation of Y6-PAs in solution is negligible.

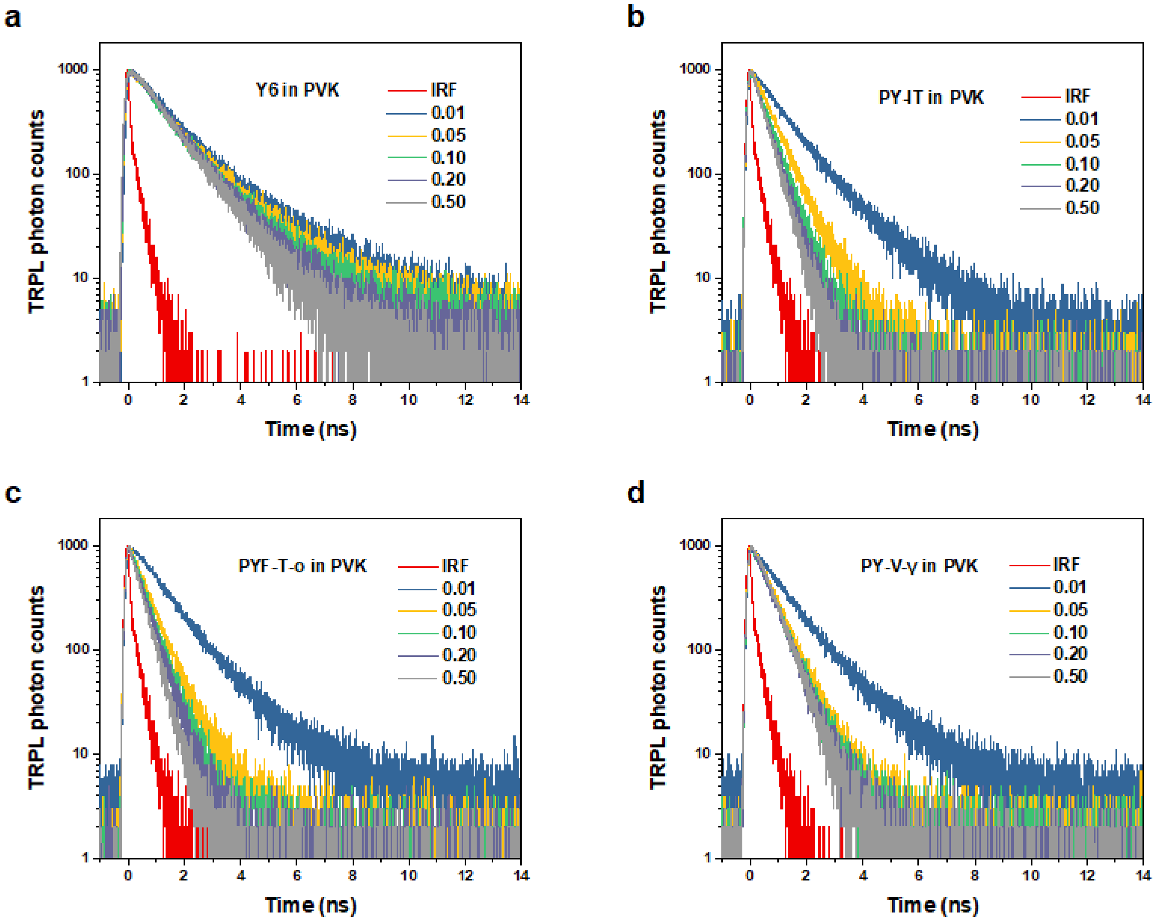

Supplementary Figure 22. TRPL profiles of Y6 (a), PY-IT (b), PYF-T-o (c), and PY-V-γ (d) dispersed in PVK with various acceptor fractions.

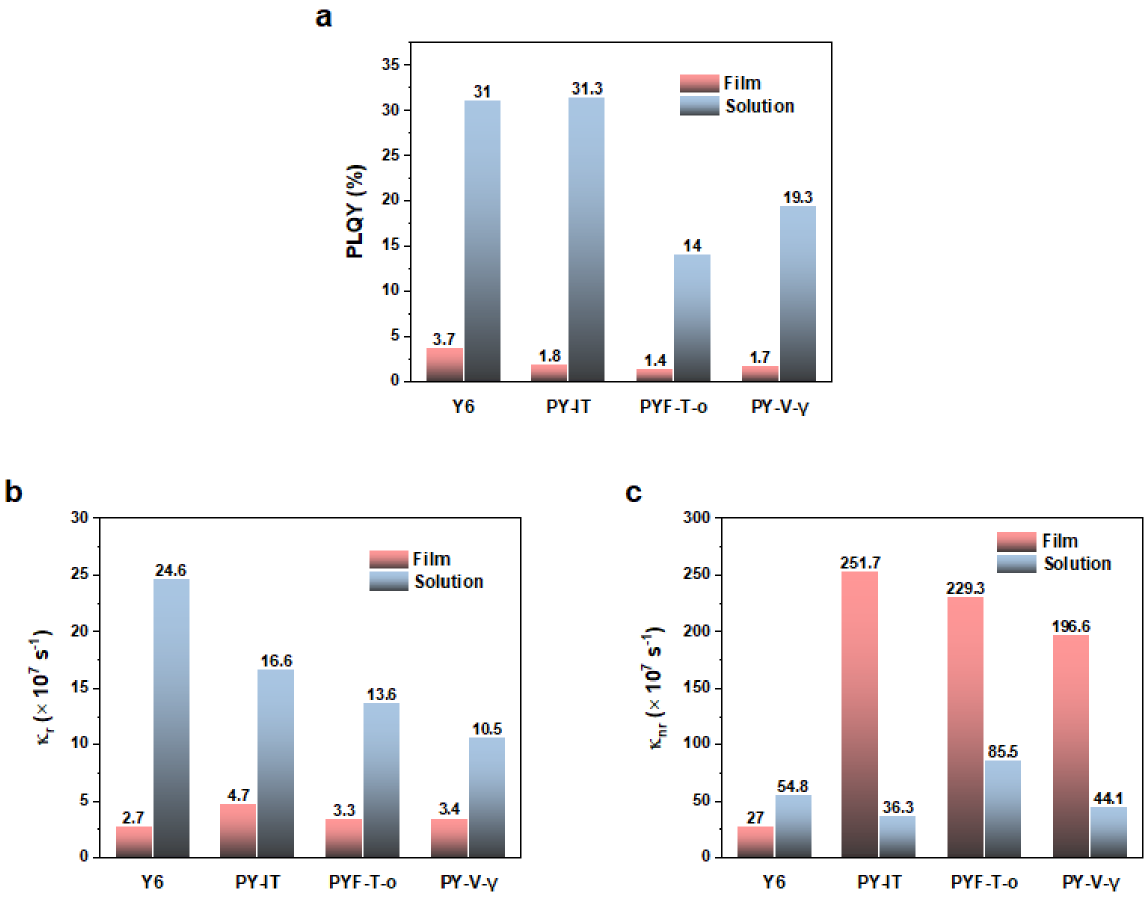

Supplementary Figure 23. PLQY (a), κ_r (b) and κ_{nr} (c) comparisons between film and solution samples.

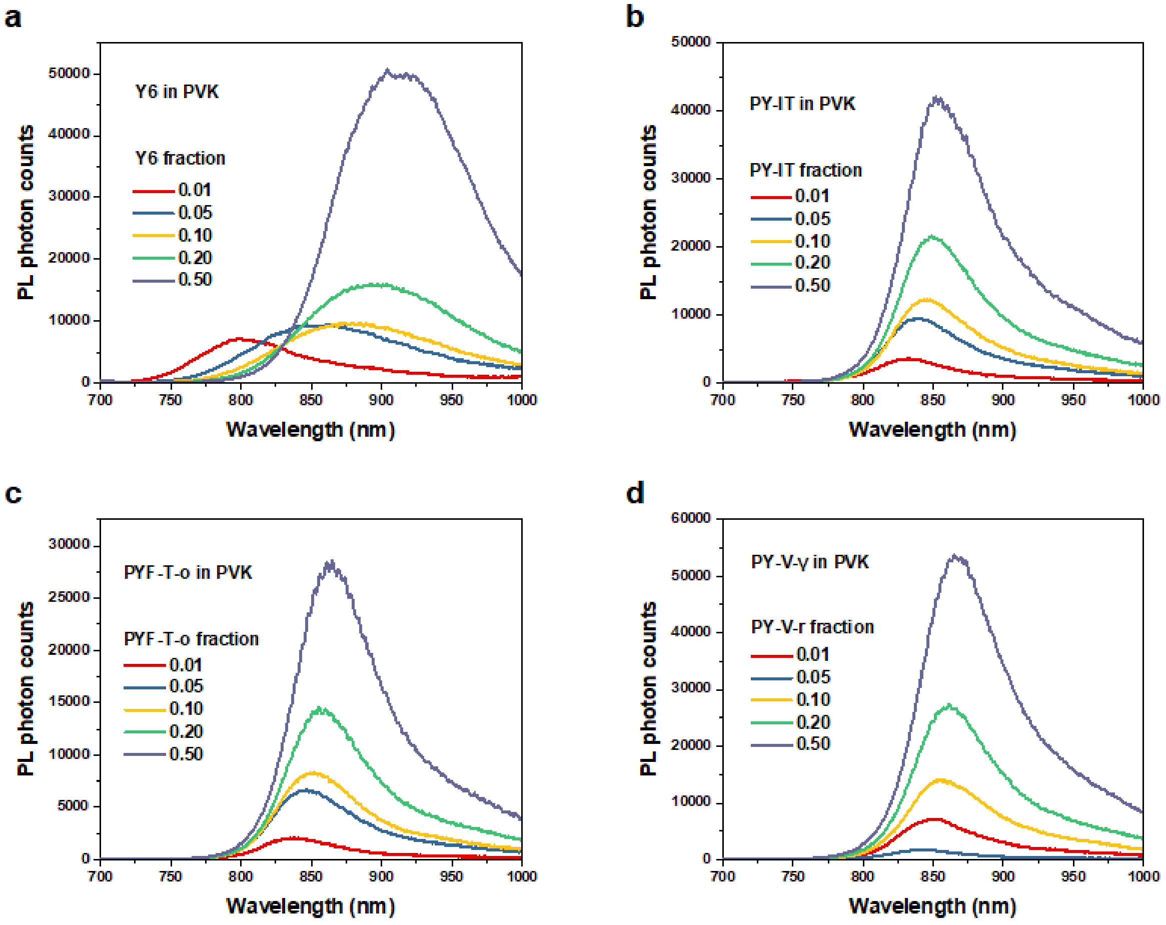

Supplementary Figure 24. PL spectra of Y6 (a), PY-IT (b), PYF-T-o (c) and PY-V-γ (d) dispersed in PVK with various acceptor fractions, excited at 680 nm.

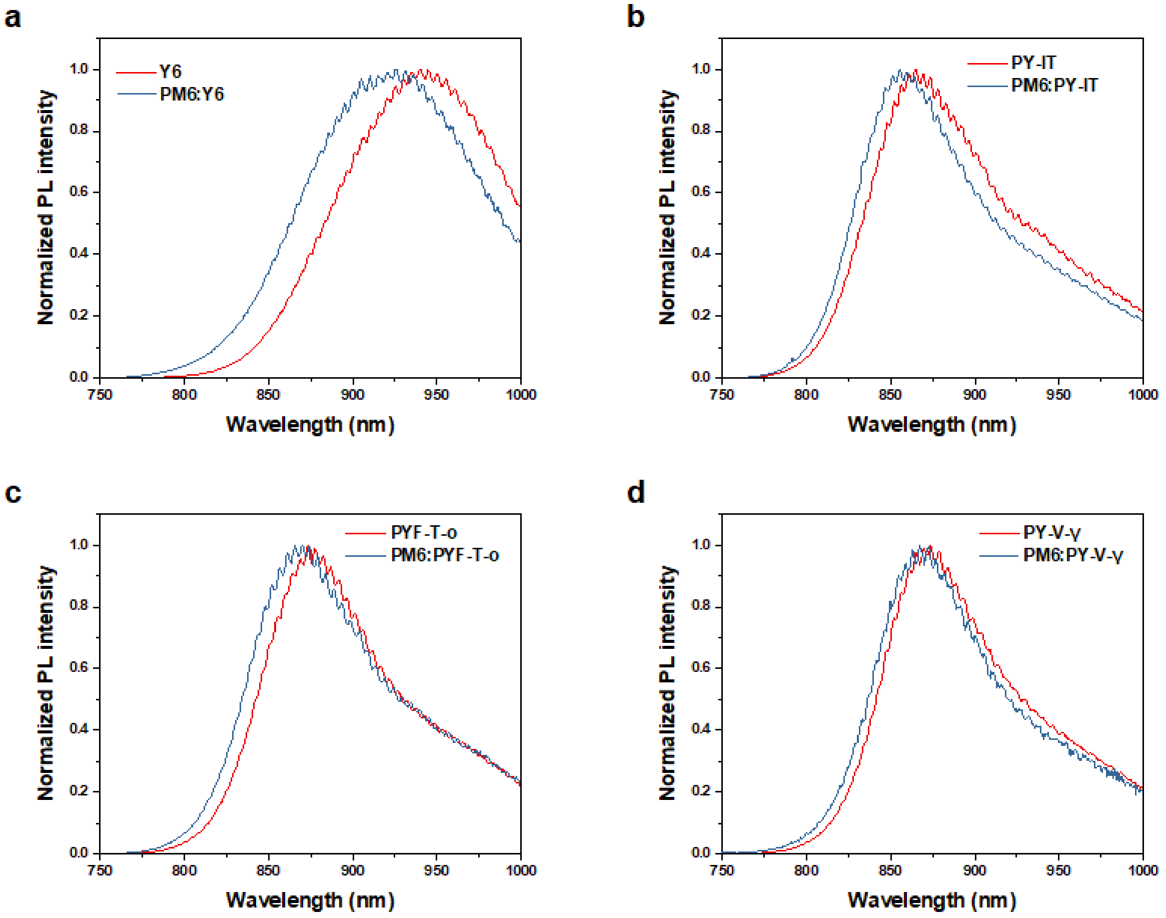

Supplementary Figure 25. PL spectra comparison between Y6 (a), PY-IT (b), PYF-T-o (c) and PY-V-γ (d) pristine acceptor and blended films, excited at 720 nm.

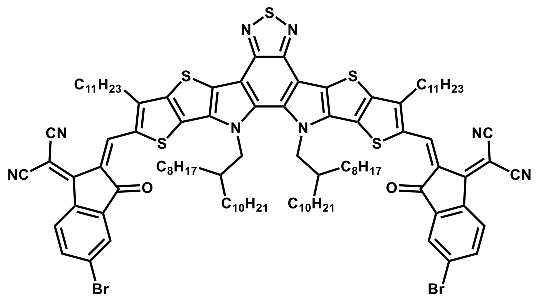

Supplementary Figure 26. Molecular structure of PY-monomer.

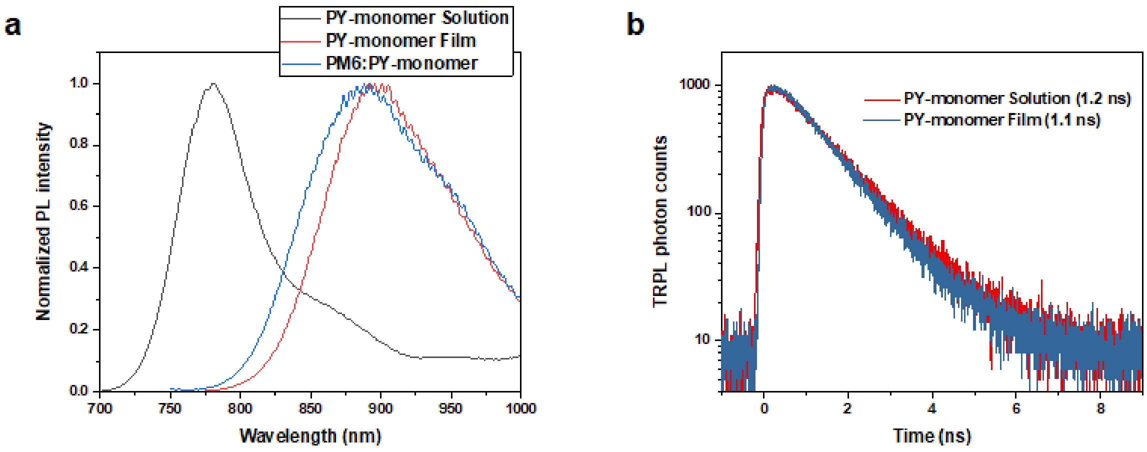

Supplementary Figure 27. (a) Normalized PL spectra for PY-monomer solution/film samples and PM6:PY-monomer blend film. (b) TRPL data for PY-monomer solution and film samples.

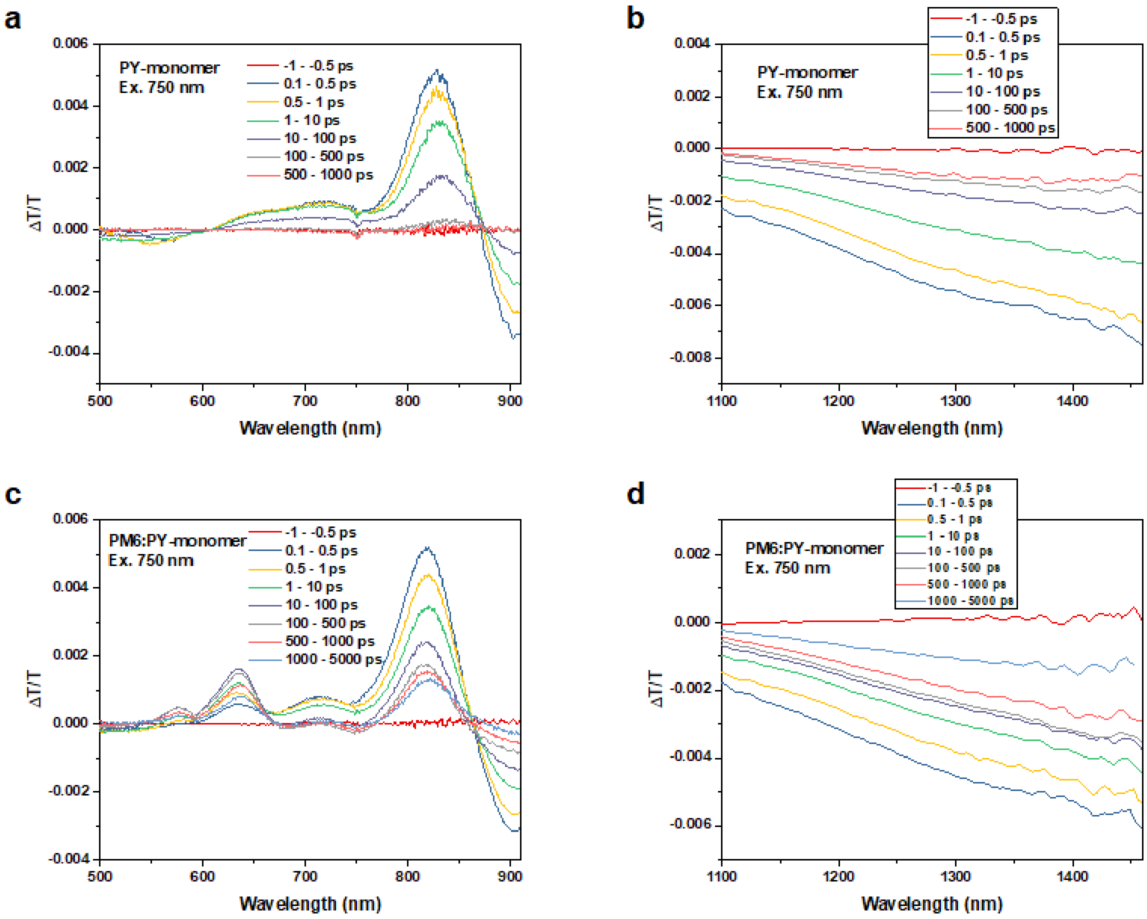

Supplementary Figure 28. TA data for neat PY-monomer film (a-b), and PM6:PY-monomer blended film (c-d).

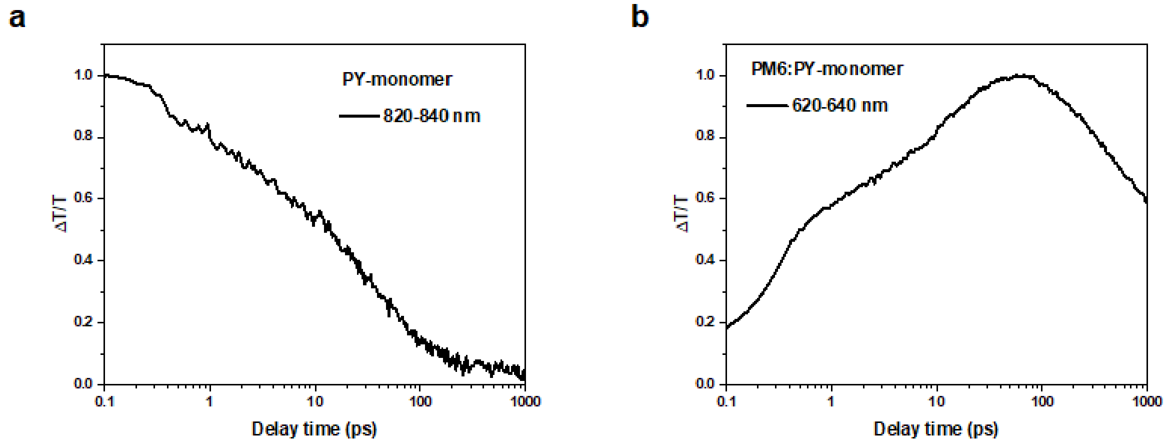

Supplementary Figure 29. Integrated decay kinetics for GSB features of PY-monomer (a) and charge generation (hole transfer) features of PM6:PY-monomer blend (b).

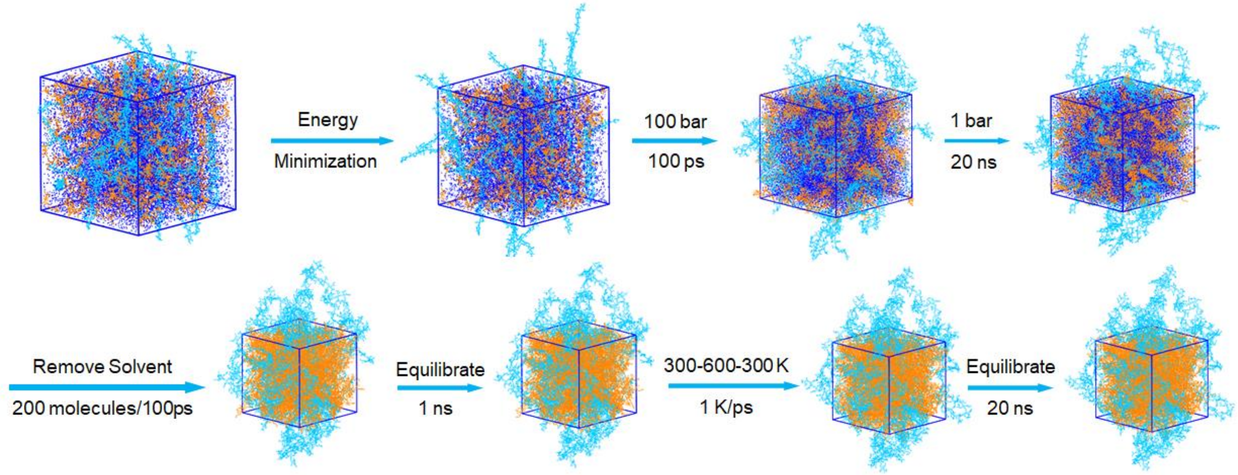

Supplementary Figure 30. Illustration of simulation process of PM6:Y6 blend which were conducted with a solvent-evaporating process.

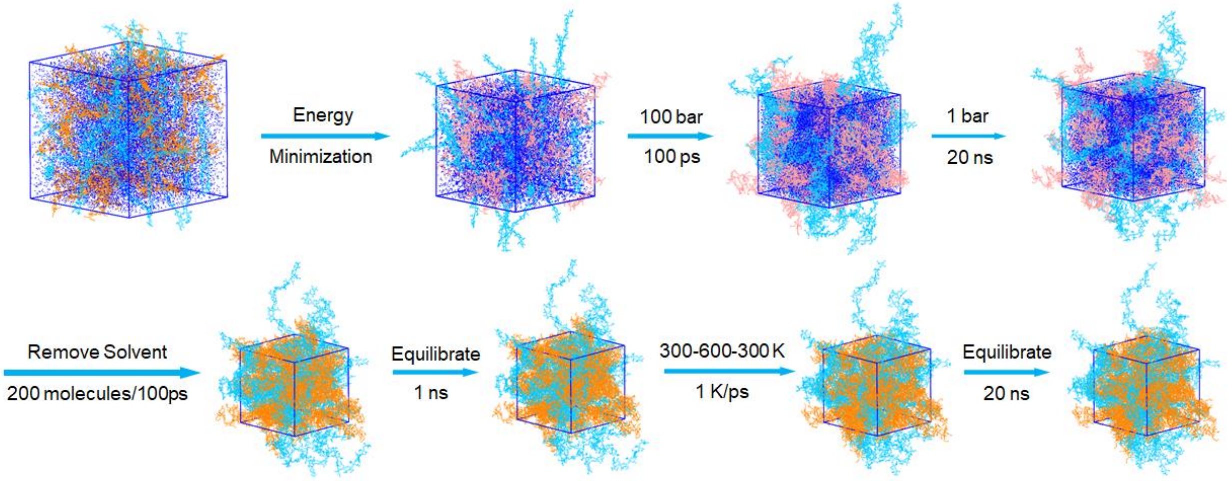

Supplementary Figure 31. Illustration of simulation process of PM6:PY-IT blend which were conducted with a solvent-evaporating process.

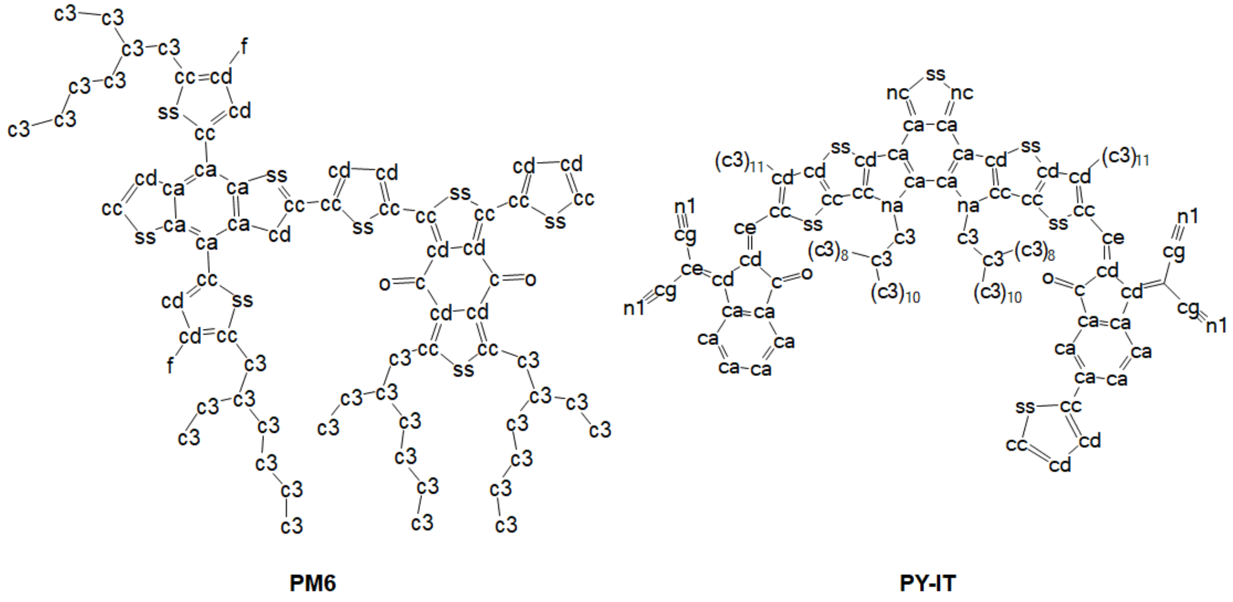

Supplementary Figure 32. Atom types used in the GAFF of repeating unit of PM6 and PY-IT.

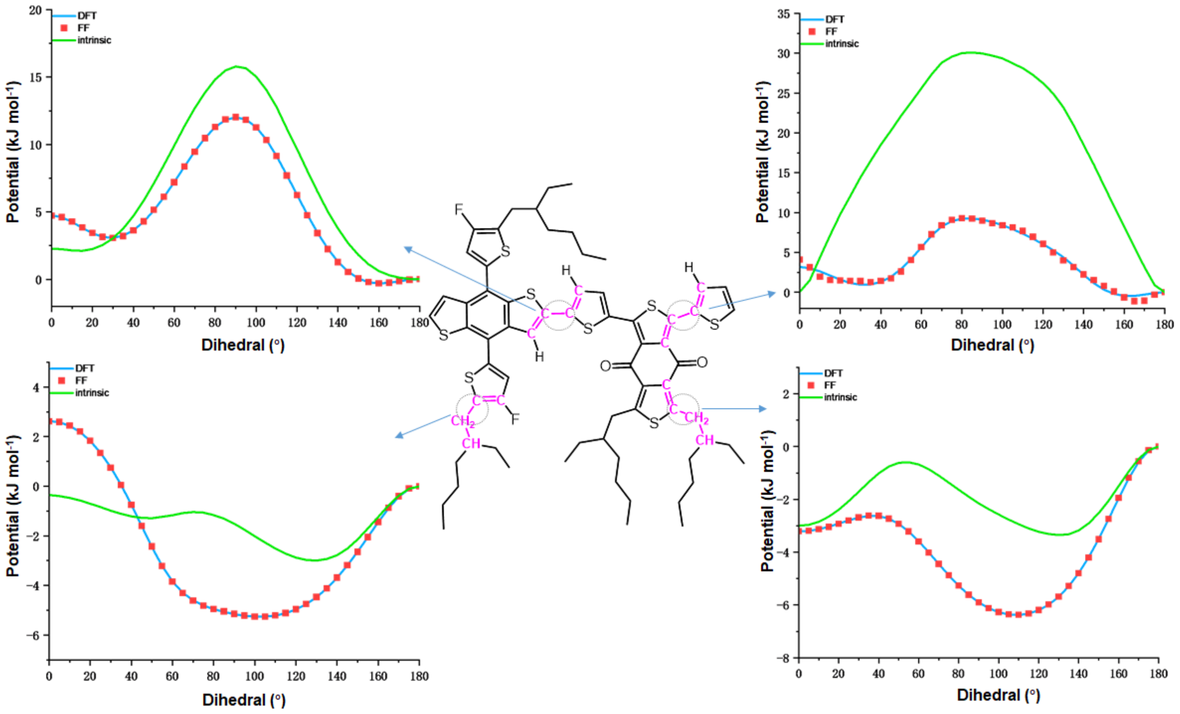

Supplementary Figure 33. Potential energy curves for the dihedral angles in repeating unit of PM6 along with the fitted intrinsic torsion potential.

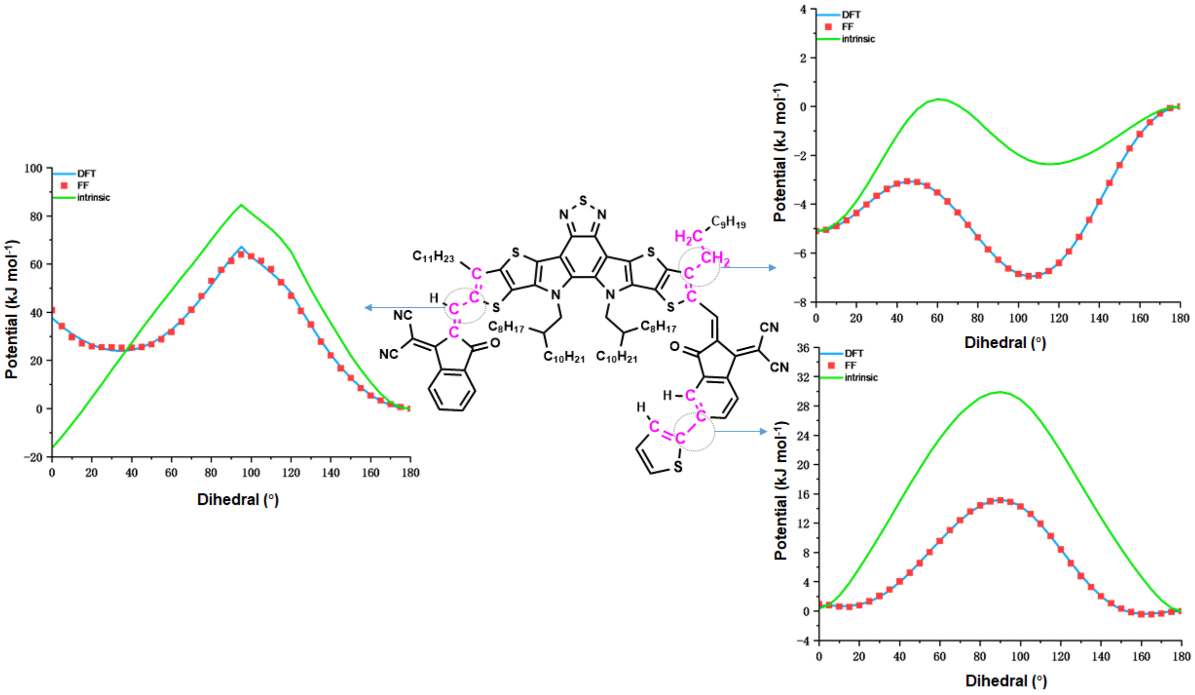

Supplementary Figure 34. Potential energy curves for the dihedral angles in repeating unit of PY-IT along with the fitted intrinsic torsion potential.

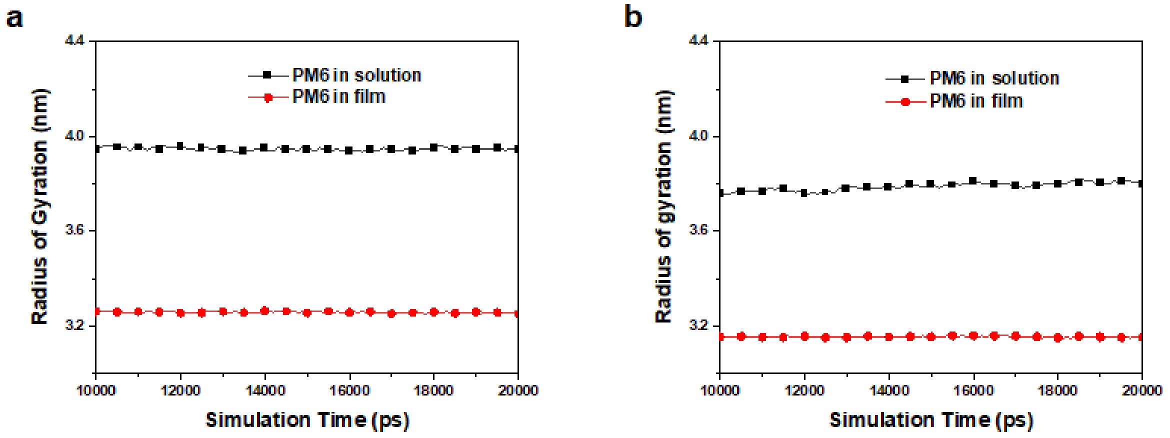

Supplementary Figure 35. Calculated R_g as a function of equilibration time of PM6 chains (both in solution and film state) in PM6:Y6 (a) and PM6:PY-IT (b) blends.

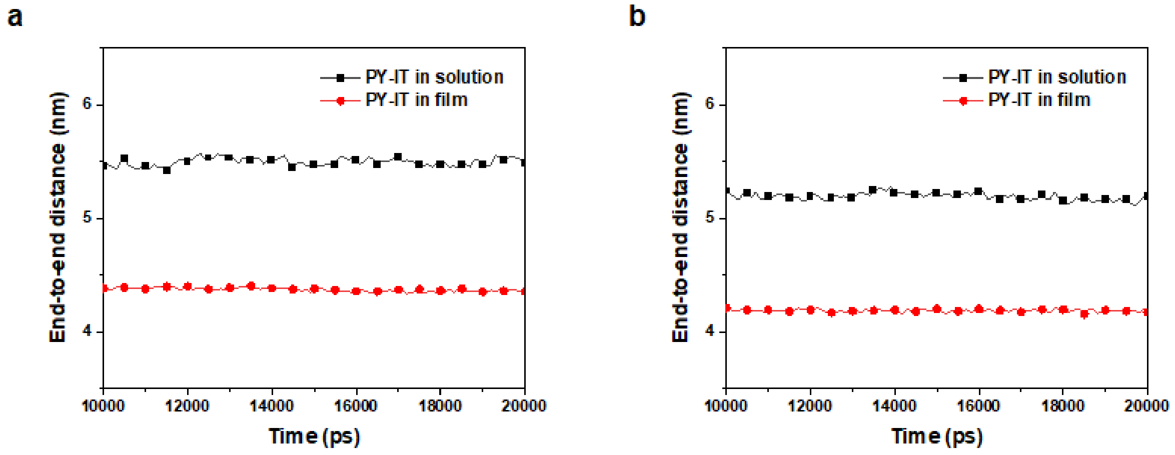

Supplementary Figure 36. Calculated end-to-end distances as a function of equilibration time of PY-IT chains (both in solution and film state) in PM6:PY-IT blend (a) and neat PY-IT phase (b).

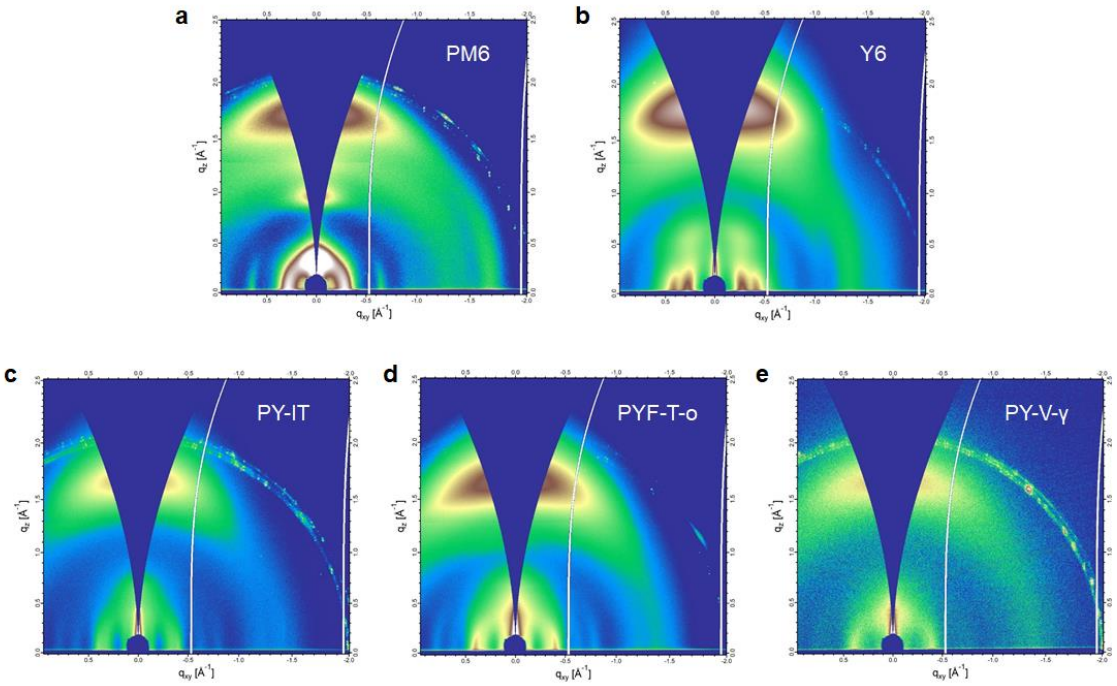

Supplementary Figure 37. GIWAXS 2D patterns of PM6 (a), Y6 (b), PY-IT (c), PYF-T-o (d) and PY-V-γ (e) neat films.

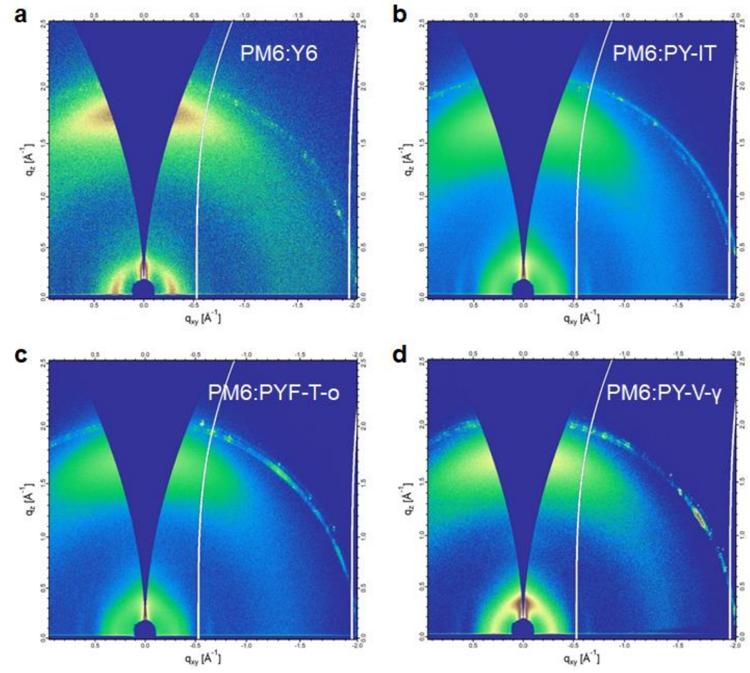

Supplementary Figure 38. GIWAXS 2D patterns of PM6:Y6 (a), PM6:PY-IT (b), PM6:PYF-T-o (c) and PM6:PY-V-γ (d) blended films.

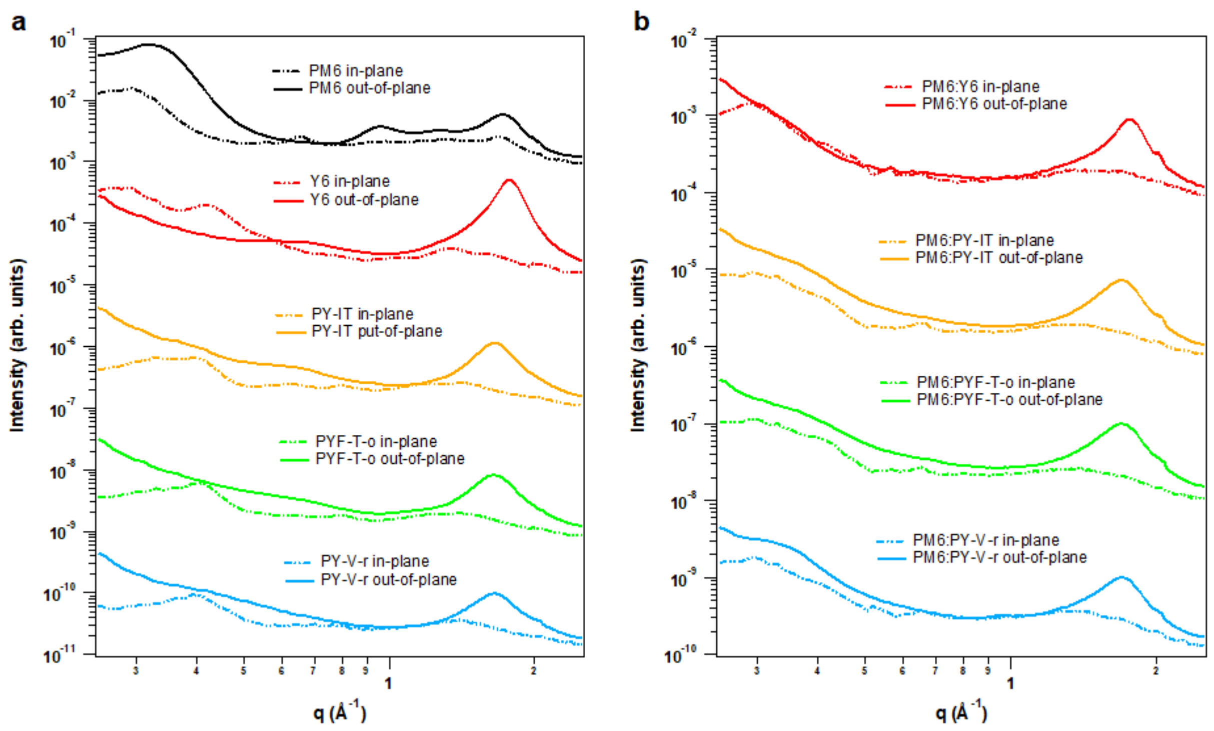

Supplementary Figure 39. GIWAXS 1D profiles along in-plane and out-of-plane directions of neat (a) and blended (b) films.

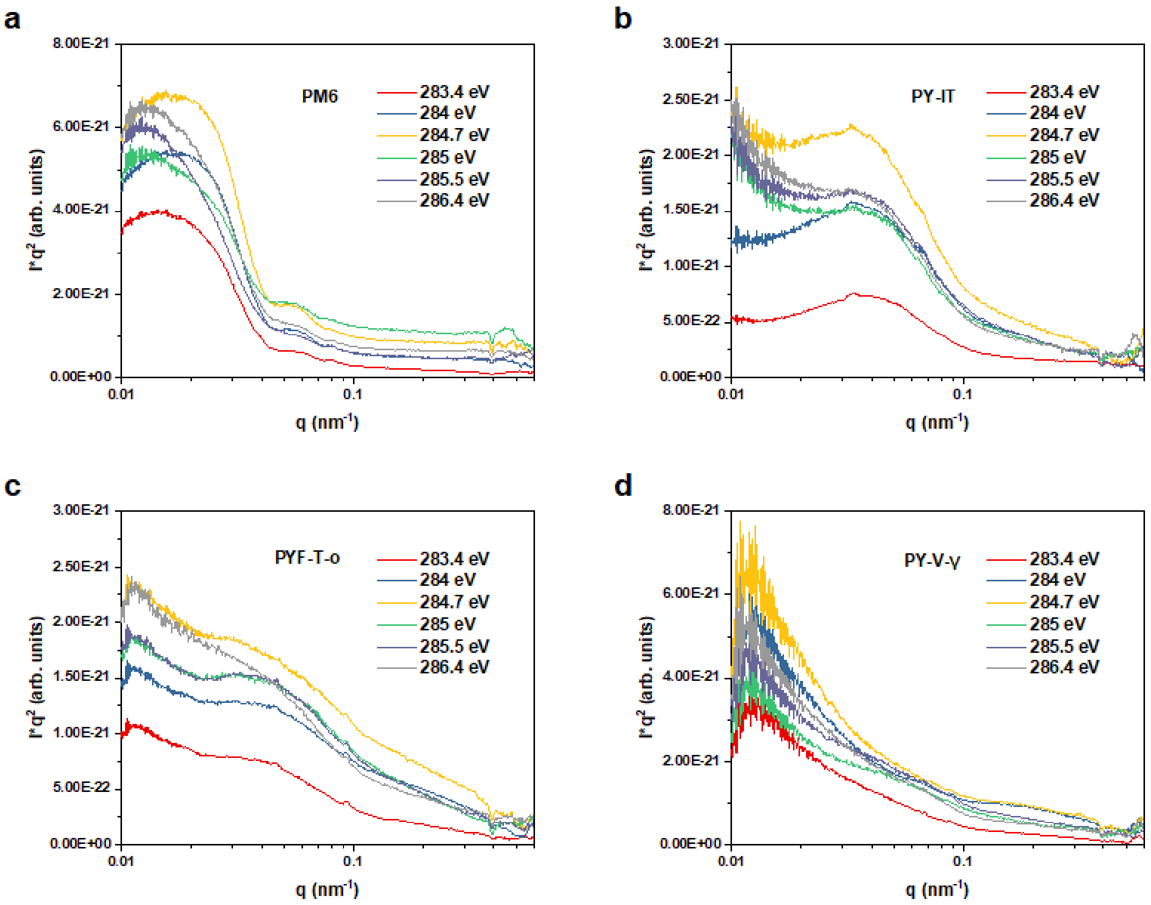

Supplementary Figure 40. R-SoXS profiles of PM6 (a), PY-IT (b), PYF-T-o (c) and PY-V-γ (d) neat films acquired with various energies.

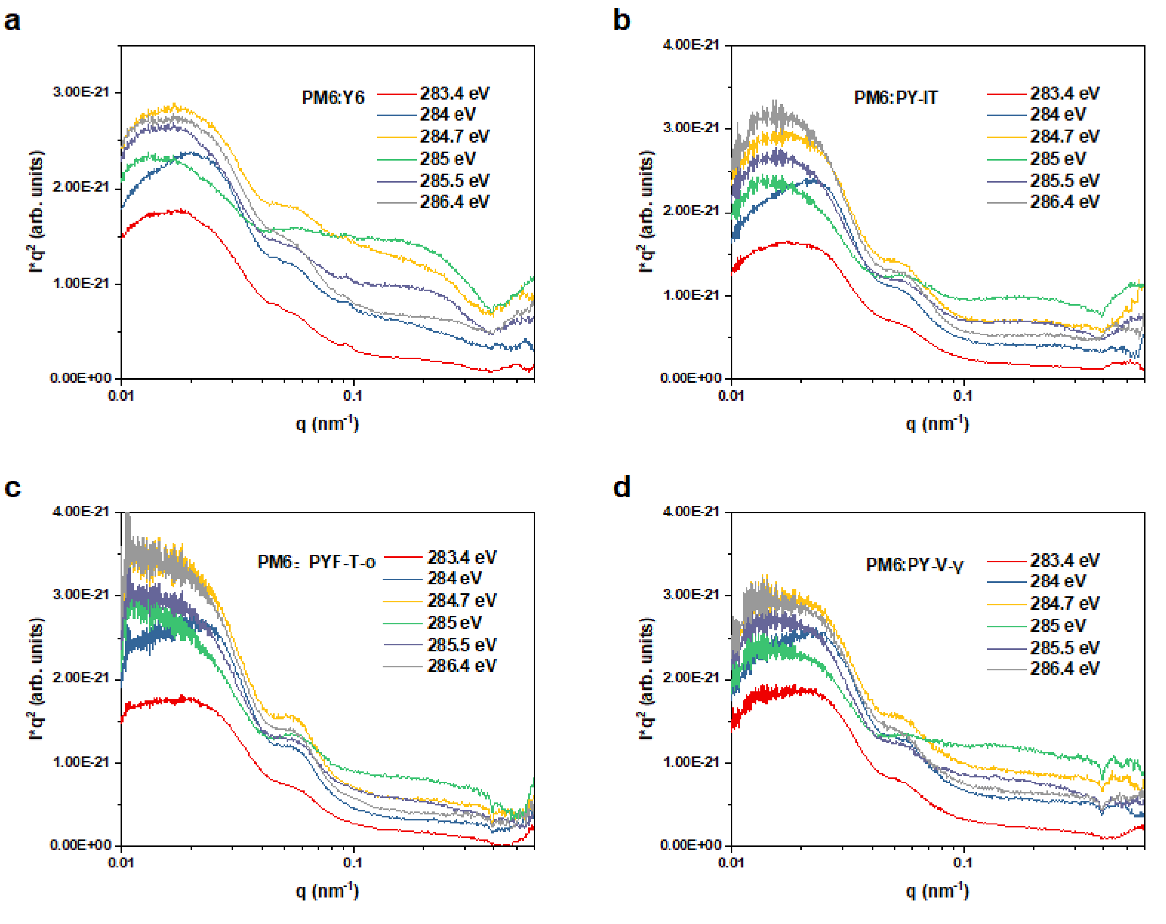

Supplementary Figure 41. R-SoXS profiles of PM6:Y6 (a), PM6:PY-IT (b), PM6:PYF-T-o (c) and PM6:PY-V-γ (d) blended films acquired with various energies.

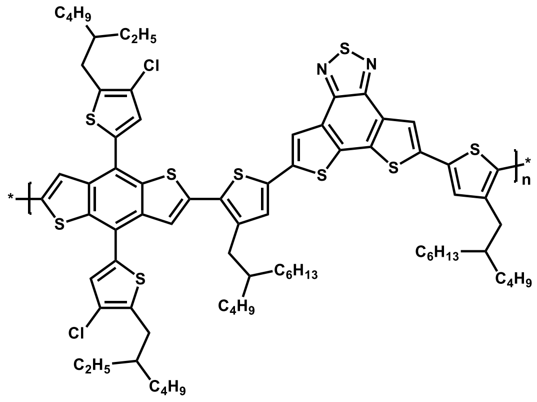

Supplementary Figure 42. Molecular structure of D18-Cl.

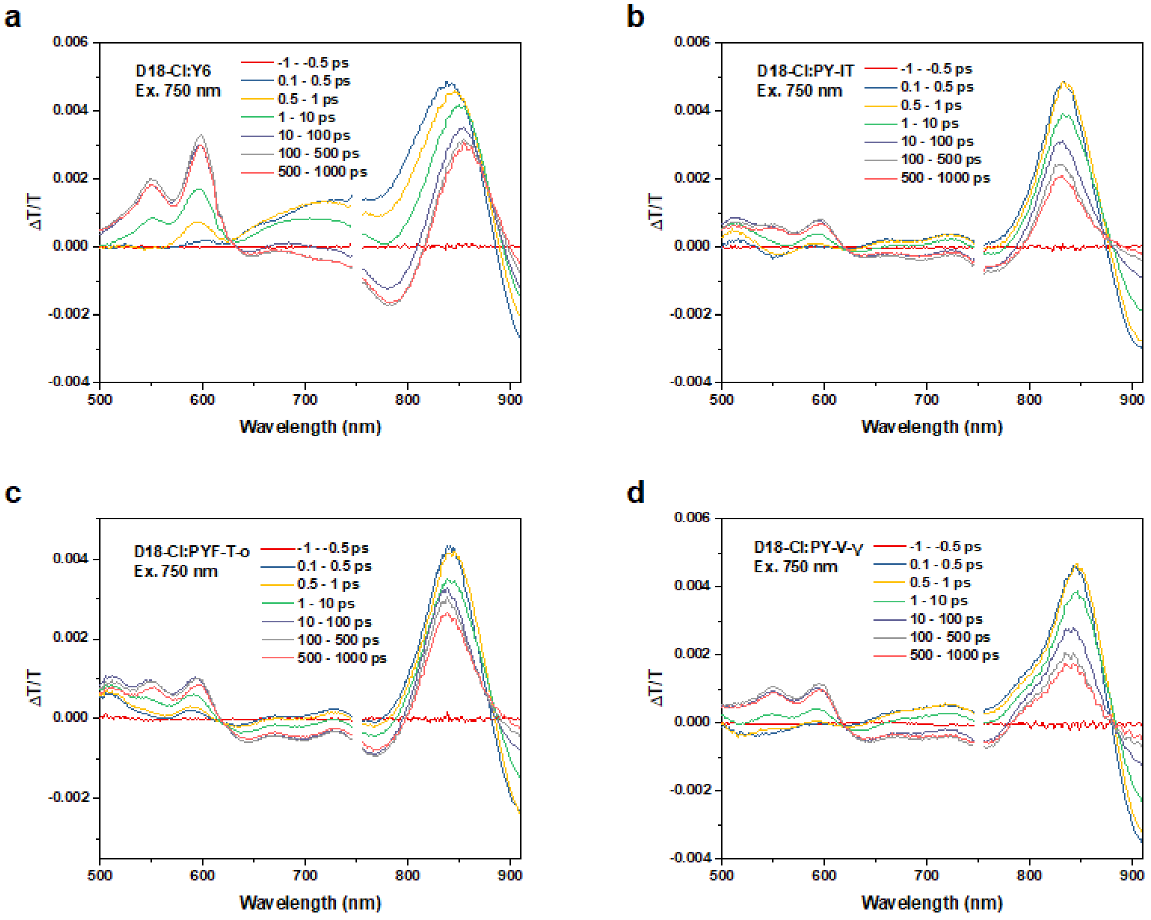

Supplementary Figure 43. TA spectra (visible region) of D18-Cl:Y6 (a), D18-Cl:PY-IT (b), D18-Cl:PYF-T-o (c), and D18-Cl: PY-V-γ (d) blends, excited at 750 nm.

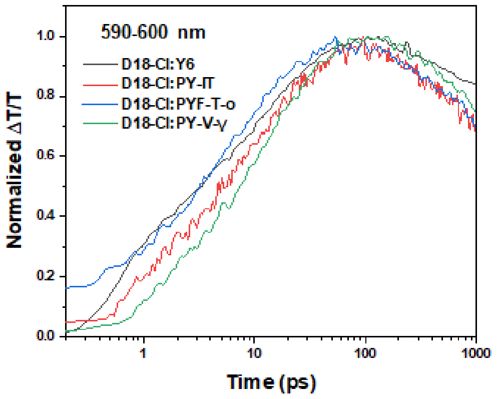

Supplementary Figure 44. Integrated normalized decay kinetics at 590-600 nm for D18-Cl blended films, excited at 750 nm.

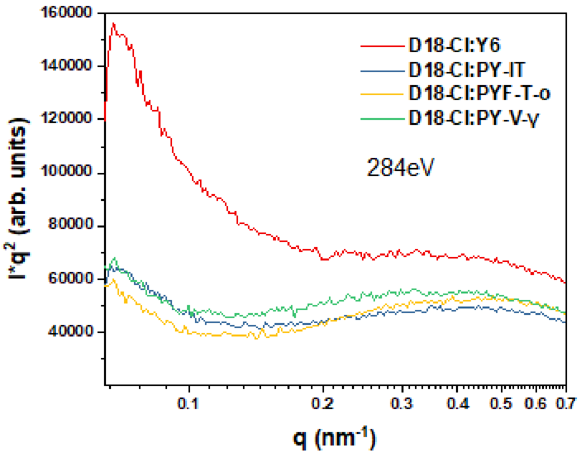

Supplementary Figure 45. Lorentz-corrected thickness-normalized R-SoXS profiles of D18-Cl:acceptor blended films, acquired at 284 eV.

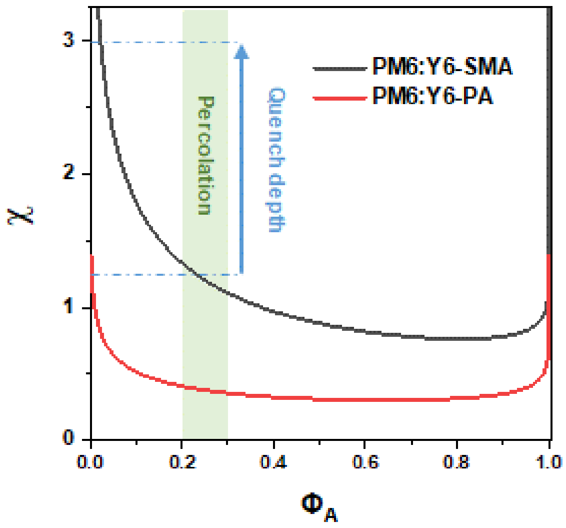

Supplementary Figure 46. Estimated binodal curves of PM6:Y6-SMA and PM6:Y6-PA based on the Flory–Huggins free energy of mixing equation for polymer solutions. “Binodal” denotes the condition at which two distinct phases may coexist.

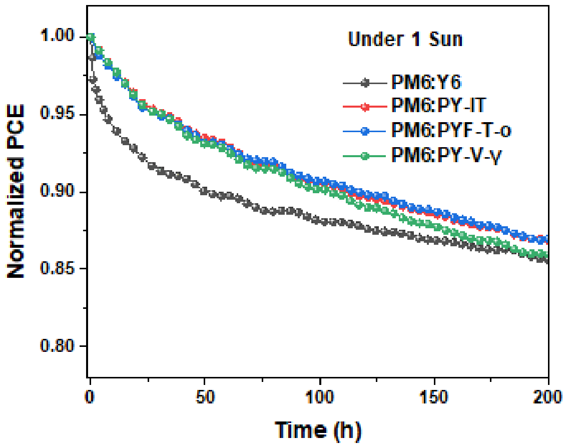

Supplementary Figure 47. Photostability of devices based on PM6:Y6, PM6:PY-IT, PM6:PYF-T-o, and PM6:PY-V-γ under 1 Sun illumination. As described in main text and Supplementary Note 3, the rapid drop in PCE in PM6:Y6 is due to device "burn-in", commonly observed in Y6-SMA-blends due to the hypo-miscible morphology. The suppressed "burn-in" in the three Y6-PA-based blends is therefore evidence for the improved blend morphology stability thanks to the increased D–A miscibility.

Supplementary Tables

Supplementary Table 1. A summary and comparison of stability (T_{80}) of Y6-SMA- and Y6-PA-based systems under 1 sun illumination (except noted otherwise).

Y6-SMA systems				
**Blend**	**T_{80} (h)**	**Publication Year**	**Ref.**	**Note**
PBDB-TF:Y6	~450	2021	10	
PM6:Y6:BTO	~250	2021	11	Ternary
PM6:Y6	~300	2022	12	
PM6:ADA:Y6	>600			Ternary
PTQ10:PTVT-T:m-BTP-PhC6	>300	2022	13	Ternary
PM6:BTP-4F-C5-16	~300	2022	14	
PM6:Y6:ZCCF3	>350	2023	15	Ternary
PM6:BTP-eC9	~660	2023	16	
Y6-PA systems				
**Blend**	**T_{80} (h)**	**Publication Year**	**Ref.**	**Note**
PM6:PYT:PY2F-T	>20000	2021	17	Ternary; Extrapolated
PBDB-T:PN-Se	>600	2021	18	
PBDB-T:PY-Se	>600	2022	19	
PBDB-T:OY3	>25000	2022	6	Oligomer; Extrapolated
PBDB-T:POY	~2400			
D18:PY-FT	>1300	2022	20	Under 65 °C
PBQx-H-TF: PBTIC-γ-TSe	~870	2022	21	
PM6:PY-1S1Se:PY-2Cl	>2000	2023	22	Ternary
PQM-Cl:PTQ10:PY-IT	>600	2023	23	Ternary

Supplementary Table 2. Summary of exciton lifetimes of acceptor solutions in CF estimated from concentration-dependent TRPL measurements.

Acceptor solution	Concentration (mg mL^{-1})	Exciton lifetime (ns)
Y6	0.001	1.19
	0.005	1.20
	0.030	1.26
PY-IT	0.001	1.59
	0.005	1.68
	0.030	1.89

PYF-T-o	0.001	0.99
	0.005	1.01
	0.030	1.03
PY-V-γ	0.001	1.46
	0.005	1.50
	0.030	1.83

Supplementary Table 3. Summary of exciton lifetimes of PVK-dispersed acceptors with various acceptor factions.

Acceptor in PVK	Acceptor fraction	Exciton lifetime (ns)
Y6	0.01	1.50
	0.05	1.49
	0.1	1.48
	0.2	1.43
	0.5	1.31
PY-IT	0.01	1.42
	0.05	0.77
	0.1	0.66
	0.2	0.56
	0.5	0.49
PYF-T-o	0.01	1.31
	0.05	0.67
	0.1	0.60
	0.2	0.54
	0.5	0.45
PY-V-γ	0.01	1.42
	0.05	0.85
	0.1	0.76
	0.2	0.74
	0.5	0.65

Supplementary Table 4. Summary of radiative recombination rate (κ_r) and non-radiative recombination rate (κ_{nr}) along with exciton lifetime (τ) and PLQY (η).

Acceptor	State	τ (ns)	η (%)	$\kappa_r \times 10^7$ s^{-1}	$\kappa_{nr} \times 10^7$ s^{-1}
Y6	Film	1.36	3.7	2.7	27.0
	Solution	1.26	31.0	24.6	54.8
PY-IT	Film	0.39	1.8	4.7	251.7
	Solution	1.89	31.3	16.6	36.3
PYF-T-o	Film	0.43	1.4	3.3	229.2
	Solution	1.03	14.0	13.6	83.5
PY-V-γ	Film	0.50	1.7	3.4	196.6
	Solution	1.83	19.3	10.5	44.1

Supplementary Table 5. Summary of the GIWAXS results ((010) peaks along out-of-plane direction), including *q* value, π–π stacking distance (*d*), full-width at half-maximum (FWHM), coherence length (L_C), for neat and blended films.

(010) peaks of neat films				
Sample	Y6	PY-IT	PYF-T-o	PY-V-γ
q (Å^{-1})	1.77	1.65	1.65	1.65
d (Å)	3.55	3.81	3.81	3.81
FWHM (Å^{-1})	0.24	0.33	0.36	0.38
L_C (Å)	23.5	17.1	15.7	14.9
(010) peaks of blended films				
Sample	PM6:Y6	PM6:PY-IT	PM6:PYF-T-o	PM6:PY-V-γ
q (Å^{-1})	1.75	1.68	1.68	1.68
d (Å)	3.59	3.74	3.74	3.74
FWHM (Å^{-1})	0.28	0.40	0.42	0.41
L_C (Å)	20.2	14.1	13.4	13.8

Supplementary Table 6. Summary of photovoltaic performance of devices based on PM6:Y6, PM6:PY-IT, PM6:PYF-T-o and PM6:PY-V-γ.

Blend	V_{OC} (V)	J_{SC} (mA cm^{-2})	FF (%)	PCE_{avg} (%)	PCE_{max} (%)
PM6:Y6	0.876 ± 0.003	25.55 ± 0.59	71.5 ± 1.6	16.09 ± 0.69	16.94
PM6:PY-IT	0.932 ± 0.002	22.14 ± 0.26	66.4 ± 0.4	13.77 ± 0.23	14.13
PM6:PYF-T-o	0.918 ± 0.007	23.59 ± 1.08	60.7 ± 2.8	13.19 ± 0.15	13.48
PM6:PY-V-γ	0.914 ± 0.003	25.12 ± 0.41	67.0 ± 1.0	15.52 ± 0.21	15.90

Device performance is averaged from 12 cells.

Supplementary References

1 Kozub, D. R. *et al.* Polymer Crystallization of Partially Miscible Polythiophene/Fullerene Mixtures Controls Morphology. *Macromolecules* **44**, 5722-5726 (2011).

2 Peng, Z., Stingelin, N., Ade, H. & Michels, J. J. A materials physics perspective on structure–processing–function relations in blends of organic semiconductors. *Nat. Rev. Mater.* **8**, 439-455 (2023).

3 Sun, G. *et al.* High performance polymerized small molecule acceptor by synergistic optimization on π-bridge linker and side chain. *Nat. Commun.* **13**, 5267 (2022).

4 Ye, L. *et al.* Miscibility-Function Relations in Organic Solar Cells: Significance of Optimal Miscibility in Relation to Percolation. *Adv. Energy Mater.* **8**, 1703058 (2018).

5 Ghasemi, M. *et al.* A molecular interaction-diffusion framework for predicting organic solar cell stability. *Nat. Mater.* **20**, 525-532 (2021).

6 Liang, Y. *et al.* Organic solar cells using oligomer acceptors for improved stability and efficiency. *Nat. Energy* **7**, 1180 (2022).

7 Azzouzi, M. *et al.* Nonradiative Energy Losses in Bulk-Heterojunction Organic Photovoltaics. *Phys. Rev. X* **8**, 031055 (2018).

8 Wöpke, C. *et al.* Traps and transport resistance are the next frontiers for stable non-fullerene acceptor solar cells. *Nat. Commun.* **13**, 3786 (2022).

9 Wang, Y. *et al.* The critical role of the donor polymer in the stability of high-performance non-fullerene acceptor organic solar cells. *Joule* **7**, 810-829 (2023).

10 Ma, L. *et al.* Completely non-fused electron acceptor with 3D-interpenetrated crystalline structure enables efficient and stable organic solar cell. *Nat. Commun.* **12**, 5093 (2021).

11 Chen, H. *et al.* A guest-assisted molecular-organization approach for >17% efficiency organic solar cells using environmentally friendly solvents. *Nat. Energy* **6**, 1045-1053 (2021).

12 Cheng, Y. *et al.* Oligomer-Assisted Photoactive Layers Enable >18 % Efficiency of Organic Solar Cells. *Angew. Chem. Int. Ed.* **61**, 202200329 (2022).

13 Ma, R. *et al.* High-Efficiency Ternary Organic Solar Cells with a Good Figure-of-Merit Enabled by Two Low-Cost Donor Polymers. *ACS Energy Lett.* **7**, 2547-2556 (2022).

14 Zhou, B. *et al.* On the Stability of Non-fullerene Acceptors and Their Corresponding Organic Solar Cells: Influence of Side Chains. *Adv. Funct. Mater.* **32**, 2206042 (2022).

15 Zhang, C. *et al.* Diffusion-Limited Accepter Alloy Enables Highly Efficient and Stable Organic Solar Cells. *Adv. Funct. Mater.* **33**, 2214392 (2023).

16 Fu, J. *et al.* 19.31% binary organic solar cell and low non-radiative recombination enabled by non-monotonic intermediate state transition. *Nat. Commun.* **14**, 1760 (2023).

17 Sun, R. *et al.* Achieving over 17% efficiency of ternary all-polymer solar cells with two well-compatible polymer acceptors. *Joule* **5**, 1548-1565 (2021).

18 Du, J. *et al.* Polymerized small molecular acceptor based all-polymer solar cells with an efficiency of 16.16% via tuning polymer blend morphology by molecular design. *Nat. Commun.* **12**, 5264 (2021).

19 Wu, Q. *et al.* Tailoring polymer acceptors by electron linkers for achieving efficient and stable all-polymer solar cells. *Natl. Sci. Rev.* **9**, nwab151 (2022).

20 Fu, H. *et al.* A Top-down Strategy to Engineer Active Layer Morphology for Highly Efficient and Stable All-polymer Solar Cells. *Adv. Mater.* **34**, 2202608 (2022).

21 Cao, C. *et al.* Quasiplanar Heterojunction All-Polymer Solar Cells: A Dual Approach to Stability. *Adv. Funct. Mater.* **32**, 2201828 (2022).

22 Sun, R. *et al.* 18.2%-efficient ternary all-polymer organic solar cells with improved stability enabled by a chlorinated guest polymer acceptor. *Joule* **7**, 221-237 (2023).

23 Ma, R. *et al.* Unveiling the Morphological and Physical Mechanism of Burn-in Loss Alleviation by Ternary Matrix Towards Stable and Efficient All-Polymer Solar Cells. *Adv. Mater.* **35**, 2212275 (2023).
