# Supplementary material for: The role of interfacial donor–acceptor percolation in efficient and stable all-polymer solar cells
Source: Nat Commun. 2024 Feb 8;15:1212. doi: 10.1038/s41467-024-45455-0 (PMC10853271; doi:10.1038/s41467-024-45455-0)
Supplement: Supplementary file 3 — Reporting Summary [file 41467_2024_45455_MOESM3_ESM.pdf]

nature portfolio

Corresponding author(s): Philip Chow

Solar Cells Reporting Summary

Nature Portfolio wishes to improve the reproducibility of the work that we publish. This form is intended for publication with all accepted papers reporting the characterization of photovoltaic devices and provides structure for consistency and transparency in reporting. Some list items might not apply to an individual manuscript, but all fields must be completed for clarity.

For further information on Nature Research policies, including our data availability policy, see Authors & Referees.

Experimental design

Please check the following details are reported in the manuscript, and provide a brief description or explanation where applicable.

1. Dimensions

Item	Yes	No	Description / Explanation
Area of the tested solar cells	[x]	[ ]	0.04 cm^2
			Explain why this information is not reported/not relevant.
Method used to determine the device area	[x]	[ ]	Controlled with an iron aperture with a 2 mm * 2 mm window, whose area is measured with a visible light microscope.
			Explain why this information is not reported/not relevant.

2. Current-voltage characterization

Item	Yes	No	Description / Explanation
Current density-voltage (J-V) plots in both forward and backward direction	[ ]	[x]	Forward direction J-V curves are measured, as it is reported in these systems backward curves are fairly similar.
Voltage scan conditions	[x]	[ ]	Forward direction scan from -1 V to 1 V, with a speed of 0.1 V/s.
			Explain why this information is not reported/not relevant.
Test environment	[x]	[ ]	Measured under room temperature in a nitrogen atmosphere glove box with oxygen and water levels are controlled under 10 ppm.
			Explain why this information is not reported/not relevant.
Protocol for preconditioning of the device before its characterization	[x]	[ ]	No encapsulation or anti-reflection coatings are applied before characterization.
			Explain why this information is not reported/not relevant.
Stability of the J-V characteristic	[ ]	[x]	*Provide a description of the method used. The stability of the J-V characteristic can be verified with time evolution of the maximum power point or with the photocurrent at maximum power point; see ref. 5 for details.*
			Explain why this information is not reported/not relevant.

3. Hysteresis or any other unusual behaviour

Item	Yes	No	Description / Explanation
Description of the unusual behaviour observed during the characterization	[ ]	[x]	*Provide a description of hysteresis or any other unusual behaviour observed during the characterization.*
			No unusual behavior was observed during the characterization.
Related experimental data	[ ]	[x]	*Provide a description of the related experimental data.*
			No related data applicable.

4. Efficiency

Item	Yes	No	Description / Explanation
External quantum efficiency (EQE) or incident photons to current efficiency (IPCE)	[ ]	[x]	*Provide a description of the technique used.*
			No EQE/IPCE data needed in this work.
A comparison between the integrated response under the standard reference spectrum and the response measure under the simulator	[ ]	[x]	*State where this information can be found in the text.*
			No EQE/IPCE data needed in this work.

April 2023

For tandem solar cells, the bias illumination and bias voltage used for each subcell

- [ ] Yes — *Provide a description of the measurement conditions.*
- [x] No — No tandem cells reported.

5. Calibration

Light source and reference cell or sensor used for the characterization

- [x] Yes — A standard silicon solar cell is applied.
- [ ] No — *Explain why this information is not reported/not relevant.*

Confirmation that the reference cell was calibrated and certified

- [x] Yes — Silicon reference cell (91150V, Newport Corporation).
- [ ] No — *Explain why this information is not reported/not relevant.*

Calculation of spectral mismatch between the reference cell and the devices under test

- [ ] Yes — *Provide a value of the spectral mismatch and/or a description of how it has been taken into account in the measurements.*
- [x] No — Spectra mismatch is not considered.

6. Mask/aperture

Size of the mask/aperture used during testing

- [x] Yes — 0.04 cm^2
- [ ] No — *Explain why this information is not reported/not relevant.*

Variation of the measured short-circuit current density with the mask/aperture area

- [x] Yes — Within 2% variance.
- [ ] No — *Explain why this information is not reported/not relevant.*

7. Performance certification

Identity of the independent certification laboratory that confirmed the photovoltaic performance

- [ ] Yes — *Identify the independent certification laboratory.*
- [x] No — Not certified with independent laboratory. Not reporting recording efficiencies.

A copy of any certificate(s)

- [ ] Yes — *Certificate copies should be provided in the Supplementary information. Please state the supplementary item number.*
- [x] No — Not applicable

8. Statistics

Number of solar cells tested

- [x] Yes — 12 devices for each system.
- [ ] No — *Explain why this information is not reported/not relevant.*

Statistical analysis of the device performance

- [x] Yes — Averaged from 12 datasets and standard deviation is calculated and shown in Table S6.
- [ ] No — *Explain why this information is not reported/not relevant.*

9. Long-term stability analysis

Type of analysis, bias conditions and environmental conditions

- [x] Yes — J-V curves (-0.1-1 V) were continuously measured under a LED light source that matched with the AM 1.5G spectrum. The test is under MPP mode, in a nitrogen atmosphere glove box without preconditions. Temerature in the chamber is controlled below 35 degree C.
- [ ] No — *Explain why this information is not reported/not relevant.*
